# Five new subterranean amphipods of the genus *Pseudocrangonyx* from Korea (Crustacea, Amphipoda, Pseudocrangonyctidae)

**DOI:** 10.3897/zookeys.970.55035

**Published:** 2020-09-21

**Authors:** Tae Won Jung, Jong Guk Kim, Min-Seop Kim, Seong Myeong Yoon

**Affiliations:** 1 National Institute of Ecology, Yeongyang 36531, South Korea National Institute of Ecology Yeongyang South Korea; 2 Korea Institute of Ocean Science & Technology, Busan 49111, South Korea Korea Institute of Ocean Science & Technology Busan South Korea; 3 National Marine Biodiversity Institute of Korea, Seocheon 33662, South Korea National Marine Biodiversity Institute of Korea Seocheon South Korea; 4 Department of Biology, Chosun University, Gwangju 61452, South Korea Chosun University Gwangju South Korea

**Keywords:** Korea, new species, pseudocrangonyctid, stygobiotic amphipods, taxonomy

## Abstract

Although the majority of the species belonging to the genus *Pseudocrangonyx* Akatsuka & Komai, 1922 are found among the subterranean fauna of eastern Asia, the taxonomic knowledge is very poor and only four species have been recorded in Korea. In this study, the morphology of the stygobitic pseudocrangonyctid amphipods from Korean subterranean waters was examined and five new species were identified: *Pseudocrangonyx concavus***sp. nov.** has a characteristic emarginated posteroventral margin of epimeral plate 3; *Pseudocrangonyx gracilipes***sp. nov.** differs from other pseudocrangonyctids by the slender and elongated pereopods and more produced posterodistal corner of epimeral plate 3. *Pseudocrangonyx crassus***sp. nov.** shows the expanded peduncular articles and a reduced flagellum of antenna 2. *Pseudocrangonyx minutus***sp. nov.** is distinguished by more reduced pleopod articles compare to other pseudocrangonyctids. *Pseudocrangonyx villosus***sp. nov.** has the more setose bases of pereopods 3 and 4. Detailed descriptions and illustrations are presented for these five new species.

## Introduction

Stygobitic amphipods of the genus *Pseudocrangonyx* are the dominant crangonyctoids in subterranean waters and springs of the east Asia region including the Far East of Russia, eastern China, Japan, and the Korean peninsula (Labay 2001, Sidorov and Gontcharov 2013, Tomikawa et al. 2016, Zhao and Hou 2017, Lee et al. 2018, 2020, Tomikawa and Nakano 2018).

To date, 27 described species of the genus *Pseudocrangonyx* have been reported in the east Asia region (Horton et al. 2020). Thirteen species have been described from the Far East of Russia, including *P. bohaensis* (Derzhavin, 1927), *P. levanidovi* Birstein, 1955, *P. camtschaticus* Birstein, 1955, *P. birsteini* Labay, 1999, *P. relicta* Labay, 1999, *P. susanaensis* Labay, 1999, *P. korkishkoorum* Sidorov, 2006, *P. febras* Sidorov, 2009, *P. elenae* Sidorov, 2011, *P. kseniae* Sidorov, 2012, *P. holsingeri* Sidorov & Gontcharov, 2013, *P. sympatricus* Sidorov & Gontcharov, 2013, and *P. tiunovi* Sidorov & Gontcharov, 2013 (Derzhavin 1927, Birstein 1955, Labay 1999, Sidorov 2006, 2009, 2011, 2012, Sidorov and Gontcharov 2013). The seven species known in Japan are *P. kyotonis* Akatsuka & Komai, 1922, *P. shikokunis* Akatsuka & Komai, 1922, *P. yezonis* Akatsuka & Komai, 1922, *P. gudariensis* Tomikawa & Sato, 2016, *P. akatsukai* Tomikawa & Nakano, 2018, *P. komaii* Tomikawa & Nakano, 2018, and *P. uenoi* Tomikawa, Abe & Nakano, 2019 (Akatsuka and Komai 1922, Tomikawa and Sato 2016, Tomikawa and Nakano 2018, Tomikawa et al. 2019). The four species known in China are *P. manchuricus* Oguro, 1938, *P. asiaticus* Uéno, 1934, *P. cavernarius* Hou & Li, 2003, and *P. elegantulus* Hou, 2017 (Uéno 1934, Oguro 1938, Hou and Li 2003, Hou 2017). In Korea, only four species have been reported: *P. asiaticus* Uéno, 1934, *P. coreanus* Uéno, 1966, *P. daejeonensis* Lee, Tomikawa, Nakano & Min, 2018, and *P. joolaei* Lee, Tomikawa, Nakano & Min, 2020 (Uéno 1934, 1966, Lee et al. 2018, 2020).

The first record of Korean pseudocrangonyctid amphipods was *P. asiaticus* from Dongryonggul Cave in North Korea (Uéno 1940). Later, Uéno (1966) examined other specimens collected from several caves in central South Korea and reported that *P. asiaticus* and *P. coreanus* were both present with variations in the character states of the several appendages such as maxilla 1, antennal flagellum, uropod 3, and telson.

Recently, several morphological and molecular phylogenetic studies of this genus have shown a high species diversity of the genus *Pseudocrangonyx* and considered *P. asiaticus* and *P. coreanus* to be a species complex (Sidorov and Gontcharov 2013, Tomikawa et al. 2016, Lee et al. 2018, 2020, Tomikawa and Nakano 2018). In this respect, the authors carefully examined the morphologies of the Korean pseudocrangonyctid specimens and found five new species: *Pseudocrangonyx concavus* sp. nov., *Pseudocrangonyx gracilipes* sp. nov., *Pseudocrangonyx crassus* sp. nov., *Pseudocrangonyx minutus* sp. nov., and *Pseudocrangonyx villosus* sp. nov. This paper presents the detailed descriptions and illustrations of these species.

## Materials and methods

The collected specimens were initially fixed in 80% ethyl alcohol in the field and then preserved in 95% ethyl alcohol after sorting in the laboratory. Specimens were stained with lignin pink before dissection. Appendages were dissected in petri dishes or excavated microscopic slides filled with a mixed solution of glycerol-ethanol using dissecting forceps and needles under a stereomicroscope (Discovery V8; ZEISS, Oberkochen, Germany) and mounted onto temporary slides using glycerol. To prepare illustrations, pencil drawings were made under a light microscope (ECLIPSE 80*i*; Nikon, Tokyo, Japan) with the aid of a drawing tube. These drawings were then scanned, digitally inked, and arranged on digital plates using the methods described by Coleman (2003, 2009). All dissected appendages and remaining bodies of type specimens were preserved in 95% ethanol. Once examined, specimens were deposited in the collection of the National Institute of Biological Resources (**NIBR**) of Korea.

## Systematic accounts

### Order Amphipoda Latreille, 1816

#### Suborder Senticaudata Lowry & Myers, 2013

**Superfamily Crangonyctoidea Bousfield, 1973**

**Family Pseudocrangonyctidae Holsinger, 1989**

**Genus *Pseudocrangonyx* Akatsuka & Komai, 1922**

##### Pseudocrangonyx concavus sp. nov.

Taxon classificationAnimaliaAmphipodaPseudocrangonyctidae

ED9C198D-D719-5A26-B556-3158F6A50E85

http://zoobank.org/C27927BA-4CC7-46B2-8875-42A550E18543

Figs 1 , 2 , 3 , 4

###### Korean name.

O-mok-nal-gae-dong-gul-yeop-sae-u, new

###### Type locality.

Hwansangul Cave, Samcheok-si, Gangwon-do, South Korea; 37°20'55"N, 129°04'45"E.

**Material examined.** Type material. ***Holotype***: 1 female, 9.2 mm, NIBRIV0000862807. ***Paratypes***: 4 specimens, NIBRIV0000872407. All type materials were collected from the type locality on 19 Jan 2001 by YG Choi.

Additional material. 1 female, 8.9 mm, NIBRIV0000872408; 1 male, 5.5 mm, NIBRIV0000872409, collected from the type locality on 13 Jan 2010 by YG Choi.

###### Etymology.

The specific name originates from the Latin word *concavus* meaning concave, hollow. This name refers to the shape of posteriorly emarginated ventral margin of epimeral plate 3.

###### Diagnosis.

Maxilla 1 inner lobe with five plumose setae on apical margin; 2^nd^ palp article with eight robust setae along distomedial and apical margins. Maxilla 2 inner lobe with one oblique row of seven plumose setae on surface; outer lobe apical margin with three plumose setae medially. Pereopod 5 coxa anterior lobe expanded ventrally (1.00 × longer than wide), margin lined with seven simple setae; basis expanded and subrectangular (0.55 × wider than long). Pereopod 6 coxa anterior lobe 0.62 × as long as that of pereopod 5, with one seta only on ventral margin; basis expanded. Sternal gills present from pereonites 2–5 (1+2+1+1 in formulae). Epimeral plate 2 ventral margin with two submarginal setae anteriorly, posterodistal corner slightly notched bearing one elongate setae. Epimeral plate 3 posterodistal corner without notch, rounded, ventral margin with three submarginal setae anteriorly, posteriorly emarginated. Uropod 1 inner ramus with one elongate seta on ventral margin subproximally. Uropod 2 peduncle 0.62 × as long as that of uropod 1.

###### Description.

**Figure 1.**
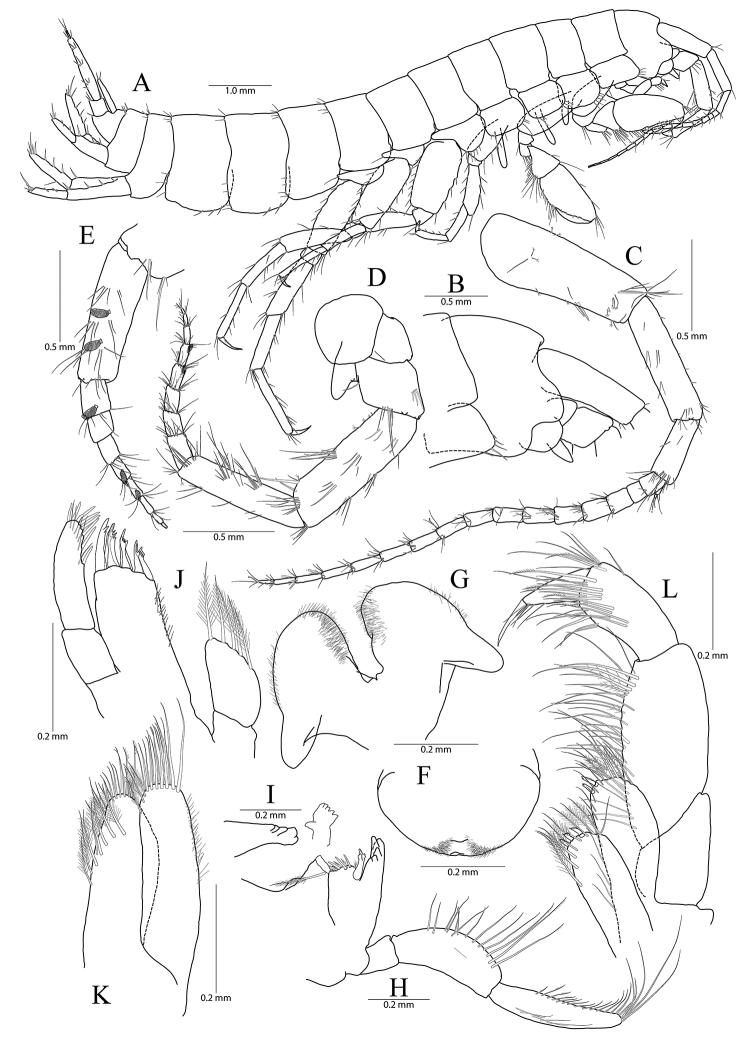
*Pseudocrangonyx concavus* sp. nov. Holotype: female, NIBRV0000862807, 9.2 mm, from Hwansangul Cave, South Korea. **A** habitus **B** head **C** antenna 1 **D, E** antenna 2, lateral (**D**) and medial (**E**) **F** upper lip **G** lower lip **H** right mandible **I** incisor and lacinia mobilis of left mandible **J** maxilla 1 **K** maxilla 2 **L** maxilliped.

Holotype female: Body (Fig. 1A) approximately 9.2 mm long.

Head (Fig. 1B) as long as pereonite 1; rostrum reduced, without minute setae; lateral cephalic lobe anteriorly expanded, apex rounded, slightly dilated anteroventrally; antennal sinus not deep; eye absent.

Antenna 1 (Fig. 1C) 0.51 × as long as body; peduncle 1^st^–3^rd^ articles length ratio of 1.00 : 0.76 : 0.40; 1^st^ article stout, with one pair of robust setae on posterior margin; accessory flagellum bi-articulate, last article very reduced, with one pair of aesthetascs distally; flagellum 1.15 × as long as peduncles, composed of 16 articles, calceoli absent, aesthetascs present on the 9^th^–11^th^, 13^th^, 14^th^, and last articles.

Antenna 2 (Fig. 1D, E) 0.59 × as long as antenna 1; antennal cone developed, apex rounded; peduncle 4^th^ and 5^th^ articles length ratio of 1.00 : 0.89; 4^th^ article margins parallel, 0.27 × wider than long, posterior margin with one row of setae at the middle and one elongate seta subdistally, 5^th^ article 0.75 × wider than 4^th^ article, two calceoli present on medial surface; flagellum composed of eight articles, 1.21 × as long as peduncle 5^th^ article, single calceoli present on 1^st^, 4^th^, and 5^th^ articles medially, aesthetascs absent.

Upper lip (Fig. 1F) anteriorly rounded, apex covered with minute setae, slightly notched.

Lower lip (Fig. 1G) inner lobes indistinct; outer lobes covered with minute setae; mandibular processes developed.

Mandibles (Fig. 1H, I) incisor 5-dentate on both sides; lacinia mobilis tri-cuspidate (each finely dentate) on right side and bi-furcate (one 5-dentate and the other not dentate) on left side; four raker setae present on both sides; molar process columnar, triturative, with one plumose seta on right side only; palp tri-articulte; 2^nd^ article convex and with 14 setae medially; 3^rd^ article subfalcate, 1.31 × as long as 2^nd^ article, lined with 19 setae from medial margin to apex.

Maxilla 1 (Fig. 1J) inner lobe subrhomboid, with five plumose setae on apical margin; outer lobe with seven dentate robust setae; palp bi-articulate, 2^nd^ article apex slightly exceeding apical setae of outer plate, with eight robust setae along distomedial and apical margins, with one oblique row of seven setae subdistally.

Maxilla 2 (Fig. 1K) inner lobe slightly shorter but wider than outer lobe, with one oblique row of seven plumose setae on surface and two rows of simple setae on apical margin; outer lobe apical margin with three plumose setae medially, also with two rows of simple setae.

Maxilliped (Fig. 1L) inner lobe subrectangular, with three dentate robust setae on apex and eight plumose setae subapically; outer lobe elongate semicircular, 0.89 × as long as palp 2^nd^ article, with two dentate robust setae and six plumose setae apically; palp composed of four articles; 2^nd^ article with many setae on medial margin; 3^rd^ article slightly dilated distally, 0.60 × as long as 2^nd^ article; 4^th^ article falcate, 0.67 × as long as 3^rd^ article, apical setae 0.67 × as long as 4^th^ article.

**Figure 2.**
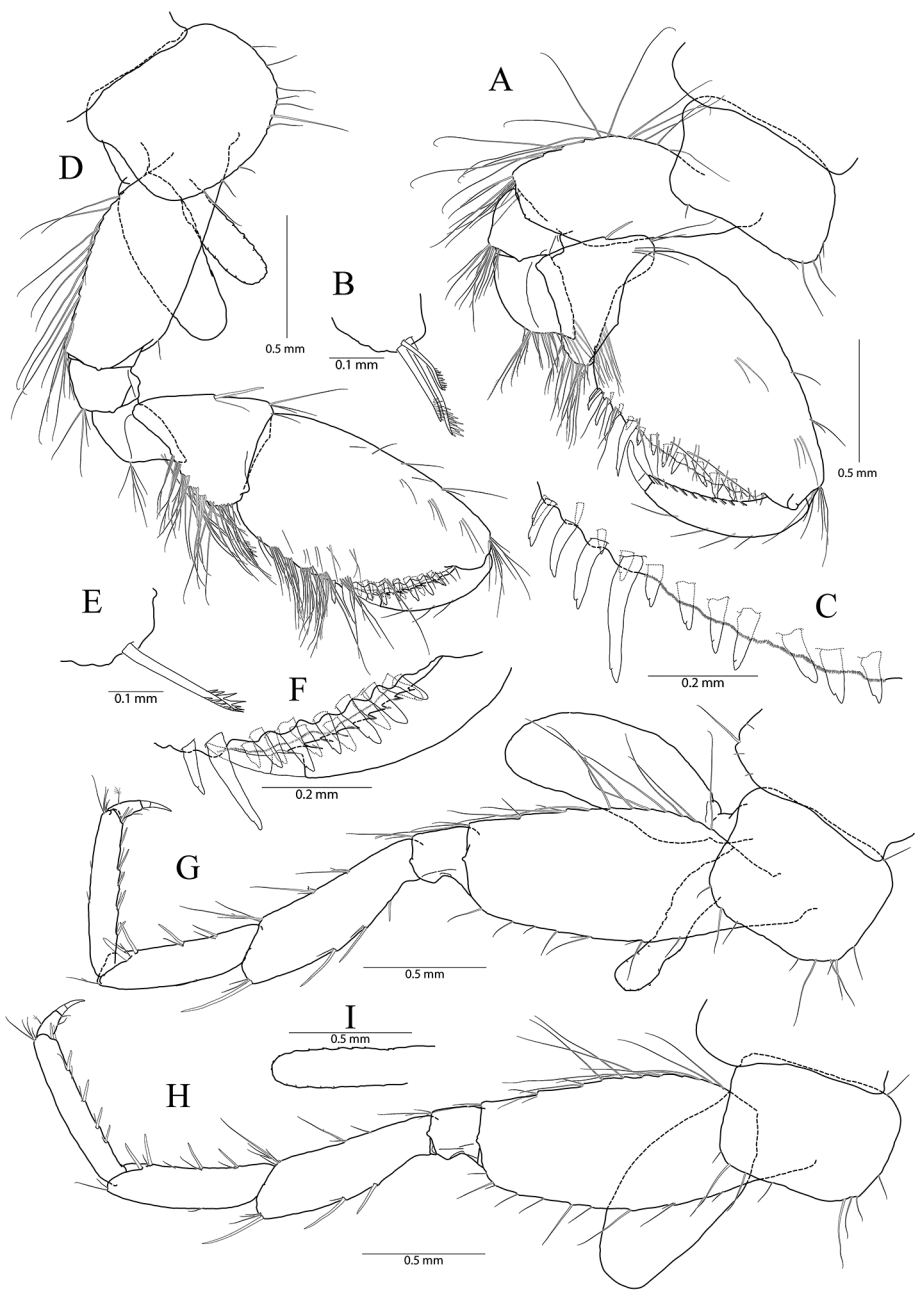
*Pseudocrangonyx concavus* sp. nov. Holotype: female, NIBRV0000862807, 9.2 mm, from Hwansangul Cave, South Korea. **A** gnathopod 1 **B** rastellate seta of gnathopod 1 **C** palm of gnathopod 1 **D** gnathopod 2 **E** rastellate setae of gnathopod 2 **F** palm of gnathopod 2 **G** pereopod 3 **H** pereopod 4 **I** oostergite of pereopod 4.

Gnathopod 1 (Fig. 2A–C) coxa subrectangular, 1.52 × wider than long, slightly produced anterodistal corner with five setae marginally, ventral margin slightly convex, with one seta posteriorly, posterior margin humped proximally; basis obtuse trapezoidal, posteriorly expanded, 0.49 × wider than long, lined with elongate simple setae posteriorly, anterior margin obliquely truncated distally, without setae; ischium 0.30 × as long as basis, with small anterior lobe; carpus 0.51× as long as basis, with one robust seta on anterior margin, carpal lobe not developed, apex rounded with three rastellate setae and many simple or serrate setae; propodus subovate, 1.30 × as long and 1.65 × as wide as basis, with five robust setae laterally along posterior margin distally and ten robust setae medially along posterior margin and palm (all medial setae small, but lateral setae larger and successively increasing distally), palm irregular, finely serrated, defined by largest lateral seta of posterior margin; dactylus as long as palm, inner margin toothed, outer margin with four setae, unguis developed.

Gnathopod 2 (Fig. 1D–F) as long as gnathopod 1; coxa 1.28 × as long as that of gnathopod 1, 1.19 × wider than long, anterior and ventral margins not produced, rounded, lined with seven setae anteriorly and three setae posteriorly, posterior margin humped proximally; coxal gill present, subovate; oostegite present, narrow, shorter than coxal gill, without marginal setae; basis subtrapezoidal, posteriorly expanded, 0.37 × wider than long, lined with elongate simple setae posteriorly, anterior margin with one seta subdistally; ischium 0.19 × as long as basis, with small anterior lobe; carpus 0.42 × as long as basis, with one pair of robust seta and simple seta on anterior margin, carpal lobe not developed, apex rounded with one rastellate seta and many simple or serrate setae; propodus trapezoidal, 0.98 × as long and 1.42 × as wide as basis, anterior margin slightly convex, posterior margin 0.49 × as long as anterior margin, lined with five clusters of elongate setae, palm irregular, finely serrated, lined with six medial and eight lateral robust setae (palm defined by two unequal setae distally among lateral setae); dactylus as long as palm, inner margin toothed, outer margin with three setae, unguis developed.

Pereopod 3 (Fig. 2G) coxa rectangular, 1.24 × wider than long, ventral margin with six anterior and three posterior setae, posterior margin slightly humped proximally; coxal gill present, subovate; oostegite present, narrow, shorter than coxal gill, without marginal setae; basis expanded, 0.80 × as wide and 2.60 × as long as coxa, width longest at proximal 1/3 and gradually diminished distally, anterior margin slightly convex, lined with simple setae, posterior margin lined with elongate setae; ischium 0.15 × as long as basis; merus anterodistally expanded, 0.31 × wider than long, 0.58 × as long as basis, anterodistal corner produced, apex blunt; carpus not expanded, 0.46 × as long as basis; propodus 0.49 × as long as basis, linear; dactylus 0.38 × as long as propodus, unguis developed.

Pereopod 4 (Fig. 2H, I) similar to pereopod 3 except that merus 0.92 × as long as that of pereopod 3; different number or position of several setae.

**Figure 3.**
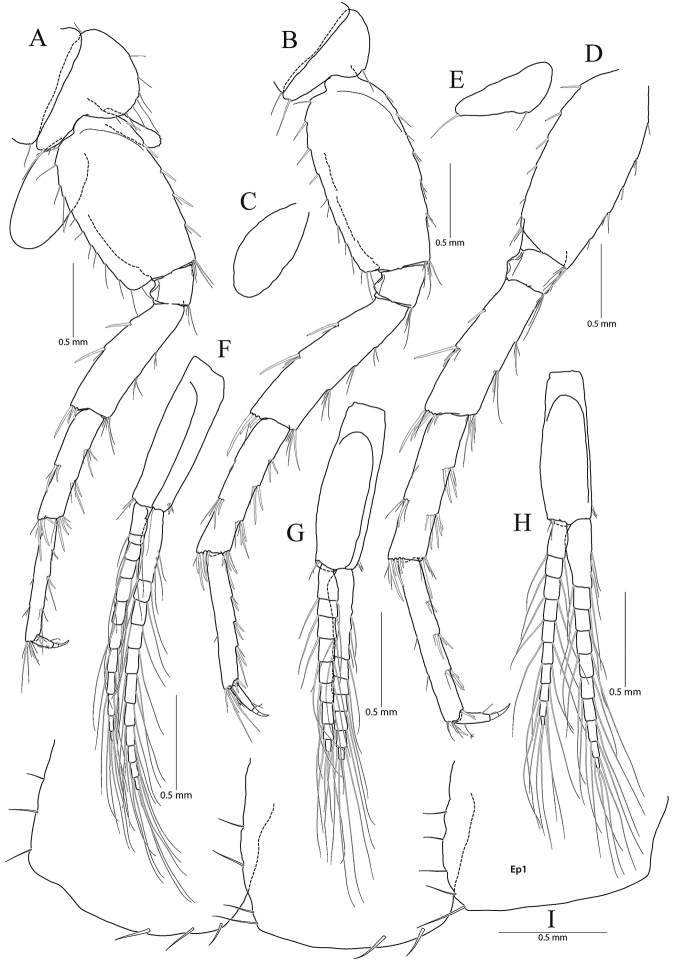
*Pseudocrangonyx concavus* sp. nov. Holotype: female, NIBRV0000862807, 9.2 mm, from Hwansangul Cave, South Korea. **A** pereopod 5 **B** pereopod 6 **C** coxal gill of pereopod 6 **D** pereopod 7 **E** coxa of pereopod **F** pleopod 1 **G** pleopod 2 **H** pleopod 3 **I** epimeral plates 1–3. Abbreviation: Ep, epimeral plate.

Pereopod 5 (Fig. 3A) coxa bilobate, anterior lobe larger than posterior lobe, expanded ventrally (1.00 × longer than wide), margin rounded, lined with seven simple setae; posterior lobe with two setae at posterior corner (ventral one very short); coxal gill present, subovate; oostegite present, narrow, shortest, without marginal setae; basis expanded, subrectangular, 0.55 × wider than long, anterior margin slightly convex, lined with three single robust setae and two pairs of robust and simple setae, with one cluster of elongate simple setae at distal corner, posterior margin lined with twelve elongate simple setae, distal corner produced forming an angle; merus posterodistally expanded, 0.42 × as wide and 0.80 × as long as basis, anterior margin with two clusters of simple setae marginally and one cluster of simple setae distally, posterior margin with one single robust seta and one pair of unequal setae marginally and one cluster of robust setae distally; carpus not expanded, 0.69 × as long as basis, anterior margin with two marginal clusters and one distal cluster of setae, posterior margin with one marginal and one distal clusters of setae; propodus linear, 1.03 × as long as carpus, anterior margin with three setal clusters (longest seta of distal cluster slightly exceeding end of propodus) and one pair of locking robust setae distally, posterior margin with one single and one pair of marginal short seta and one distal cluster of elongate setae; dactylus 0.31 × as long as propodus, unguis developed.

Pereopod 6 (Fig. 3B, C) 1.14 × as long as pereopod 5; coxa bilobate, anterior lobe 0.62 × as long as that of pereopod 5, with one seta only on ventral margin, posterior lobe expanded backward, with one seta at posterior corner; coxal gill present, subovate; oostegite absent; basis expanded, subrectangular, 1.16 × as long and 1.00 × as wide as that of pereopod 5, 0.46 × wider than long, anterior margin slightly convex, lined with three single robust setae and three clusters of robust and simple setae, with one cluster of elongate simple setae at distal corner, posterior margin lined with twelve setae, distal corner produced forming an angle; merus posterodistally expanded, 1.22 × as long as that of pereopod 5, 0.50 × as wide and 0.84 × as long as basis, anterior margin with three marginal clusters and one distal cluster of simple setae, posterior margin with two marginal clusters and one distal cluster of simple and robust setae; carpus not expanded, 0.73 × as long as basis, anterior margin with three marginal clusters and one distal cluster of simple and robust setae, posterior margin with one simple seta and one cluster of simple and robust setae marginally and one cluster of setae distally; propodus linear, 0.92 × as long as carpus, anterior margin with three setal clusters (longest seta of distal cluster not exceeding end of propodus) and one pair of locking robust setae distally, posterior margin with one marginal short seta and one distal cluster of elongate setae; dactylus 0.37 × as long as propodus, unguis developed.

Pereopod 7 (Fig. 3D, E) 1.06 × as long as pereopod 6; coxa unilobed, subtriangular, 0.77 × as long as that of pereopod 6, with one seta on ventral margin, posteriorly expanded with one seta at posterior corner;) ; coxal gill and oostegite absent; basis expanded, subrectangular, 1.00 × as long and 0.92 × as wide as that of pereopod 6, 0.42 × wider than long, anterior margin slightly convex, lined with three single setae and two clusters of setae, with one cluster of elongate simple setae at distal corner, posterior margin lined with eight setae, distal corner slightly produced but angle smaller than those of pereopods 6 and 7; merus posterodistally expanded, 0.80 × as long as that of pereopod 6, 0.54 × as wide and 0.67 × as long as basis, anterior margin with two marginal clusters and one distal cluster of simple setae, posterior margin with one single robust seta and one cluster of simple and robust setae marginally, and one cluster of simple and robust setae distally; carpus not expanded, rectangular, 0.24 × as wide as long, 1.09 × as long as merus, anterior margin with three marginal clusters and one distal cluster of simple and robust setae, posterior margin with one simple seta and one cluster of simple and robust setae marginally, and one cluster of simple and robust setae distally; propodus linear, 1.11 × as long as carpus, anterior margin with three setal clusters (longest seta of distal cluster not exceeding end of propodus) and one pair of locking robust setae distally, posterior margin with two marginal short setae and one distal cluster of setae posteriorly (shorter than those of pereopods 6 and 7); dactylus 0.38 × as long as propodus, unguis developed.

**Figure 4.**
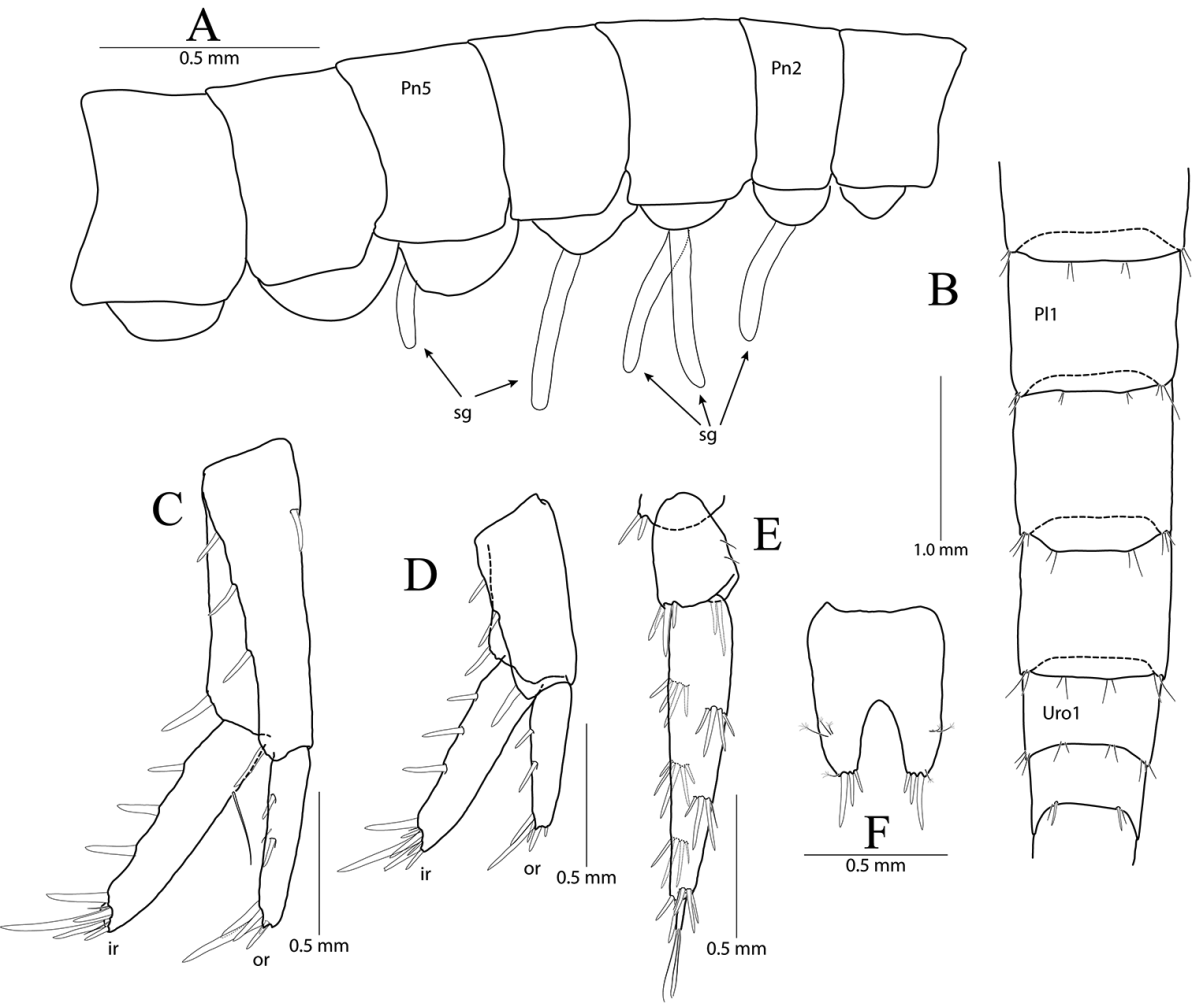
*Pseudocrangonyx concavus* sp. nov. Holotype: female, NIBRV0000862807, 9.2 mm, from Hwansangul Cave, South Korea. **A** sternal gills **B** pleonites and urosomites, dorsal **C** uropod 1 **D** uropod 2 **E** uropod 3 **F** telson. Abbreviations: Pl, pleonite; Uro, urosomite; sg, sternal gill; ir, inner ramus; and or, outer ramus.

Sternal gills (Fig. 4A) present in pereonites 2 to 5 (1+2+1+1 in formulae), narrower than oostegites, shortest in that of pereonite 5.

Epimeral plate 1 subquadrate, a little produced posteroventrally, ventral margin without setae, posterior margin with four setae, posterodistal corner slightly notched bearing one elongate seta. Epimeral plate 2 subquadrate, larger than plate 1, ventral margin with two submarginal setae anteriorly, posterior margin with three setae, posterodistal corner slightly notched bearing one elongate seta. Epimeral plate 3 posterior margin with three setae, posterodistal corner without notch, rounded, ventral margin with three submarginal setae anteriorly, posteriorly emarginated. (Fig. 3I).

Pleopod 1 (Fig. 3F) peduncle with one pair of retinaculae mediodistally and one cluster of three simple setae laterodistally; outer ramus 1.55 × as long as peduncle, composed of twelve articles; inner ramus 1.85 × as long as peduncle, composed of eleven articles (coalesced 1^st^ article as long as proximal four articles of outer ramus combined).

Pleopod 2 (Fig. 3G) peduncle 1.08 × as long as that of pleopod 1, with one pair of retinaculae mediodistally and one pair of simple setae laterodistally; outer ramus as long as peduncle, composed of nine articles; inner ramus as long as outer ramus, composed of seven articles (coalesced 1^st^ article as long as proximal four articles of outer ramus combined).

Pleopod 3 (Fig. 3H) 1.11 × as long as pleopod 2; peduncle 0.91 × as long as that of pleopod 2, with one pair of retinaculae mediodistally and one pair of simple setae laterodistally; outer ramus 1.36 × as long as peduncle, composed of ten articles; inner ramus 1.21 × as long as outer ramus, composed of ten articles (coalesced 1^st^ article 1.20 × as long as proximal two articles of outer ramus combined).

Uropod 1 (Fig. 4C) peduncle with one basofacial seta, with three marginal robust setae and one distal robust seta dorsolaterally, with one distal robust seta dorsomedially; outer ramus 0.59 × as long as peduncle, with one simple seta dorsomedially and two robust setae dorsolaterally, apical cluster composed of five robust setae; inner ramus 1.32 × as long as outer ramus, with three robust setae dorsomedially, apical cluster composed of five robust and two simple setae, with one elongate seta on ventral margin subproximally.

Uropod 2 (Fig. 4D) 0.70 × as long as uropod 1; peduncle 0.62 × as long as that of uropod 1, with one marginal seta and one distal seta on both lateral and medial margins; outer ramus 0.77 × as long as peduncle, with two robust setae dorsolaterally, apical cluster composed of five robust setae; inner ramus 1.35 × as long as outer ramus, with three robust setae dorsomedially, apical cluster composed of one simple seta and six robust setae (apex of one robust seta abnormal).

Uropod 3 (Fig. 4E) uniramous, 0.91 × as long as uropod 1; peduncle short, 0.58 × as long as that of uropod 2, with two minute setae on medial margin, with two setal clusters on mediodistal and laterodistal corners; ramus 3.1 × as long as peduncle, bi-articulate, proximal article gradually diminished in width, with three lateral and four medial clusters of setae (longest one of distal cluster exceeding distal article of ramus), distal article 0.13 × as long as proximal article, with three simple setae apically.

Telson (Fig. 4F) 0.78 × as wide as long, cleft for 43% of length, each lobe with one cluster of two or three penicillate setae dorsally, and one penicillate seta and three robust setae on apex.

###### Remarks.

*Pseudocrangonyx concavus* sp. nov. resembles *Pseudocrangonyx asiaticus* sensu Uéno (1934), but differs in the absence of calceoli in the flagellum of antenna 1 (compared to the slender calceoli present in that of *P. asiaticus*), in the presence of seven dentate setae on the apical margin of outer plate (compared to five in *P. asiaticus*) and eight setae apically on the palp of maxilla 1 (compared to four in *P. asiaticus*), by the more expanded basis of pereopods 5–7, by bearing sternal gills from pereonites 2 to 5 of 1+2+1+1 in formulae, by the presence of three and four setae on posterior margins of epimeral plates 2 and 3, respectively (compared to six and seven setae in *P. asiaticus*), by the emarginated posteroventral margin of epimeral plate 3 (not emarginated in *P. asiaticus*), by the presence of only one subproximal elongate seta on the ventral margin of the inner ramus on uropod 1 (compared to four in *P. asiaticus*), and by the longer peduncle of uropod 2 than that of *P. asiaticus* (Uéno 1934).

##### Pseudocrangonyx gracilipes sp. nov.

Taxon classificationAnimaliaAmphipodaPseudocrangonyctidae

528273D9-59D8-5BA1-B9A2-6B2C8F67061B

http://zoobank.org/57FD3300-7429-4122-9A63-828E0F4C75C4

Figs 5 , 6 , 7 , 8

###### Korean name.

Ga-neun-da-ri-dong-gul-yeop-sae-u, new

###### Type locality.

Gosugul Cave, Danyang-gun, Chungcheongbuk-do, South Korea; 36°59'12"N, 128°22'52"E.

**Material examined.** Type material. ***Holotype***: 1 female, 9.9 mm, NIBRIV0000862808, 19 Jan 2001. ***Paratype***: 1 male, 1.4 mm, NIBRIV0000872410, 11 Nov 1973; 1 male and 1 female, NIBRIV0000872411, 27 Sep 1975. All type materials were collected by YG Choi.

###### Etymology.

The specific name originates from the combination of the Latin word *gracilis*, meaning slender, thin and *pes*, meaning foot. This name refers to the shape of the elongate pereopods that are more evident than those of other pseudocrangonyctids.

###### Diagnosis.

Maxilla 1 inner lobe with four plumose setae on apical margin; 2^nd^ palp article apex with six robust setae (weakly dentate). Maxilla 2 inner lobe with one oblique row of four plumose setae on surface; outer lobe apical margin without plumose setae. Maxilliped inner lobe with three dentate robust setae on apex. Gnathopod 1 carpus 0.55 × as long as basis, with one pair of robust setae marginally on anterior margin, carpal lobe with three rastellate setae. Gnathopod 2 as long as gnathopod 1, carpal lobe with one cluster of three rastellate setae subdistally. Pereopods 5–7 articles slightly slender and elongate. Sternal gills present from pereonites 2–5 (1+1+1+1 in formulae). Epimeral plate 2 ventral margin with four submarginal setae anteriorly, posterodistal corner slightly notched bearing one elongate seta. Epimeral plate 3 posterior margin with eight setae, posterodistal corner produced backward, with weak notch, bearing one elongate seta, ventral margin with five submarginal setae anteriorly, not concave. Pleopods 1–3 peduncles with robust setae laterally. Uropod 1 inner ramus with two elongate setae subproximally on ventral margin. Telson 0.63 × as wide as long, cleft for 47% of length.

###### Description.

**Figure 5.**
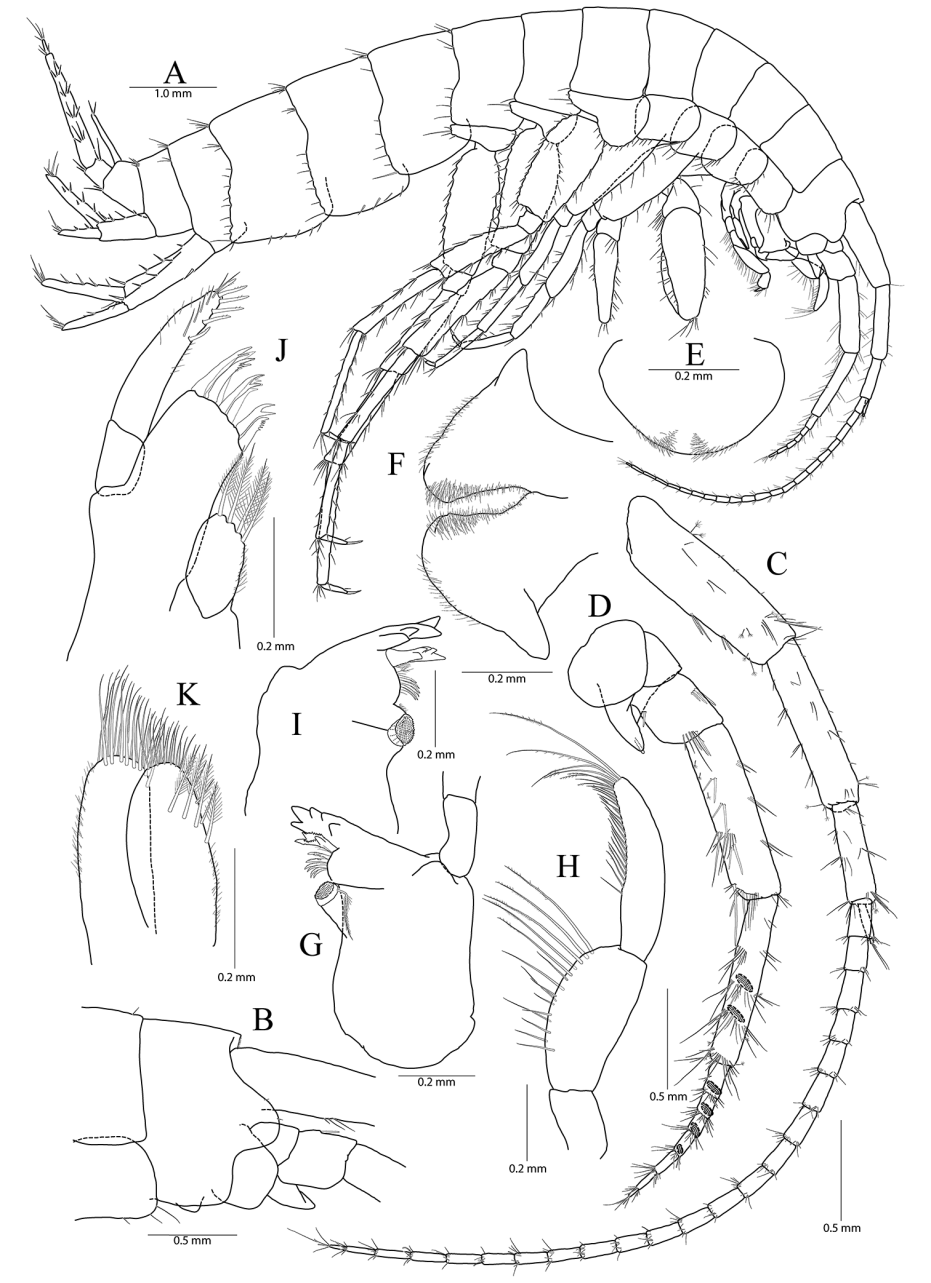
*Pseudocrangonyx gracilipes* sp. nov. Holotype: female, NIBRV0000862808, 9.9 mm, from Gosugul Cave, South Korea. **A** habitus **B** head **C** antenna 1 **D** antenna 2 **E** upper lip **F** lower lip **G, H** right mandible **I** left mandible **J** maxilla 1 **K** maxilla 2.

Holotype female: Body (Fig. 5A) approximately 9.9 mm long.

Head (Fig. 5B) 0.76 × as long as pereonite 1; rostrum reduced, with minute setae; lateral cephalic lobe anteriorly expanded, apex rounded, slightly dilated anteroventrally; antennal sinus not deep; eye absent.

Antenna 1 (Fig. 5C) 0.53 × as long as body; 1^st^–3^rd^ peduncular articles length ratio of 1.00 : 0.86 : 0.48; 1^st^ article stout, with two pairs of robust setae on posterior margin; accessory flagellum bi-articulate, last article very reduced, with one cluster of simple setae distally; flagellum 1.45 × as long as peduncles, composed of 22 articles, calceoli absent, aesthetascs present from 16^th^–20^th^ and last articles.

Antenna 2 (Fig. 5D) 0.51 × as long as antenna 1; antennal cone developed, apex rounded; 4^th^ peduncular article 0.97 × as long as 2^nd^ peduncular article of antenna 1, margins parallel, 0.25 × wider than long, densely setose with several rows or clusters of various setae; 5^th^ article 1.00 × as long and 0.67 × as wide as 4^th^ article, two calceoli present on medial surface, also densely setose with several rows or clusters of various setae; flagellum composed of seven articles, 0.91 × as long as 5^th^ peduncular article, single calceoli present from 1^st^–4^th^ articles medially, aesthetascs absent.

Upper lip (Fig. 5E) anteriorly rounded, apex covered with minute setae, not notched.

Lower lip (Fig. 5F) inner lobes indistinct; outer lobes covered with minute setae; mandibular processes developed.

Mandible (Fig. 5G–I) incisor 5-dentate on right side and 4-dentate on left side; lacinia mobilis tri-cuspidate (two finely dentate and one not dentate) on right side and bi-furcate (one 4-dentate and the other not dentate) on left side; six raker setae present on both sides; molar process columnar, triturative, with one plumose seta on right side only; palp tri-articulate; 2^nd^ article convex and with 15 setae medially; 3^rd^ article subfalcate, 1.16 × as long as 2^nd^ article, lined with 27 setae from medial margin to apex..

Maxilla 1 (Fig. 5J) inner lobe subrhomboid, with four plumose setae on apical margin; outer lobe with seven dentate robust setae; palp bi-articulate, 2^nd^ article apex obviously exceeding apical setae of outer plate, with six robust setae (weakly dentate) along distomedial to apical margins, with one oblique row of six setae subdistally.

Maxilla 2 (Fig. 5K) inner lobe slightly shorter but wider than outer lobe, with one oblique row of four plumose setae on surface and two rows of simple setae on apical margin; outer lobe apical margin also with two rows of simple setae (without plumose setae).

**Figure 6.**
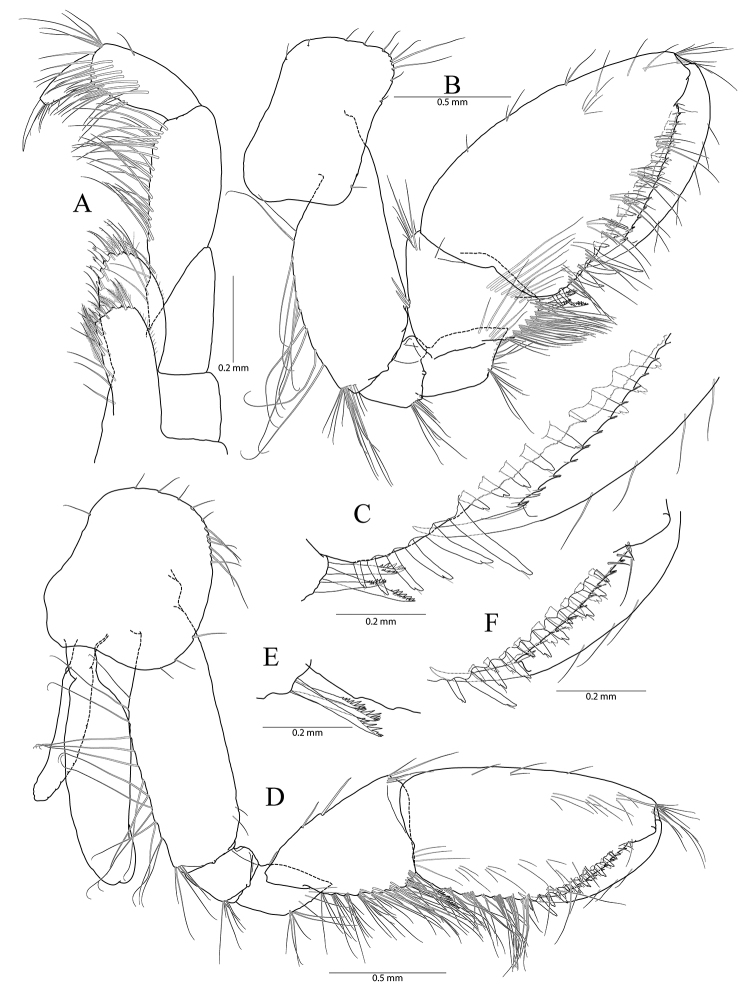
*Pseudocrangonyx gracilipes* sp. nov. Holotype: female, NIBRV0000862808, 9.9 mm, from Gosugul Cave, South Korea. **A** maxilliped **B** gnathopod 1 **C** rastellate setae and palm of gnathopod 1 **D** gnathopod 2 **E** rastellate setae of gnathopod 2 **F** palm of gnathopod 2.

Maxilliped (Fig. 6A) inner lobe subrectangular with three dentate robust setae on apex and eleven plumose setae mediodistally and subapically; outer lobe elongate semicircular, 0.62 × as long as 2^nd^ palp article, lined with four dentate robust setae (one distal seta smallest) and four plumose setae apically; palp composed of four articles; 2^nd^ article with many setae on medial margin; 3^rd^ article slightly dilated distally, 0.56 × as long as 2^nd^ article; 4^th^ article falcate, 0.61 × as long as 3^rd^ article, apical setae 0.76 × as long as 4^th^ article.

Gnathopod 1 (Fig. 6B, C) coxa subrectangular, 1.52 × wider than long, anteroventral corner slightly produced with nine setae marginally, ventral margin a little concaved, with one seta posteriorly, posterior margin humped proximally; basis obtuse trapezoidal, posteriorly expanded, 0.45 × wider than long, lined with elongate simple setae posteriorly, anterior margin obliquely truncated distally, without setae; ischium 0.27 × as long as basis, with small anterior lobe; carpus 0.55 × as long as basis, with one pair of robust setae marginally and one cluster of simple setae on anterior margin, carpal lobe not developed, apex rounded with three rastellate setae and many simple or serrate setae; propodus subtriangular, 1.38 × as long and 1.64 × as wide as basis, with six robust setae laterally along posterior margin (successively increasing distally), palm irregular, finely serrated, lined with nine robust setae medially, defined by largest lateral seta of posterior margin; dactylus exceeding largest lateral seta of posterior margin, inner margin toothed, outer margin with four setae, unguis developed.

Gnathopod 2 (Fig. 6D–F) as long as gnathopod 1; coxa 1.37 × as long as that of gnathopod 1, 1.14 × wider than long, anterior and ventral margins not produced, rounded, lined with eleven setae, ventral margin with three setae posteriorly, posterior margin humped proximally; coxal gill present, subovate; oostegite present, narrow, shorter than that of coxal gill, without marginal setae; basis subtrapezoidal, posteriorly expanded, 0.33 × wider than long, lined with elongate simple setae posteriorly, anterior margin with two setae subdistally; ischium 0.21 × as long as basis, with small anterior lobe; carpus 0.63 × as long as basis, with two robust setae on anterior margin, carpal lobe not developed, broad, with seven clusters of many simple or serrate setae, with one cluster of three rastellate setae subdistally; propodus trapezoidal, 0.93 × as long and 1.41 × as wide as basis, anterior margin slightly convex, posterior margin 0.48 × as long as anterior margin, lined with six clusters of elongate setae, palm irregular, finely serrated, lined with nine medial and eleven lateral robust setae (palm defined by two unequal setae distally among lateral setae); dactylus exceeding palm, inner margin toothed, outer margin with three setae, unguis developed.

**Figure 7.**
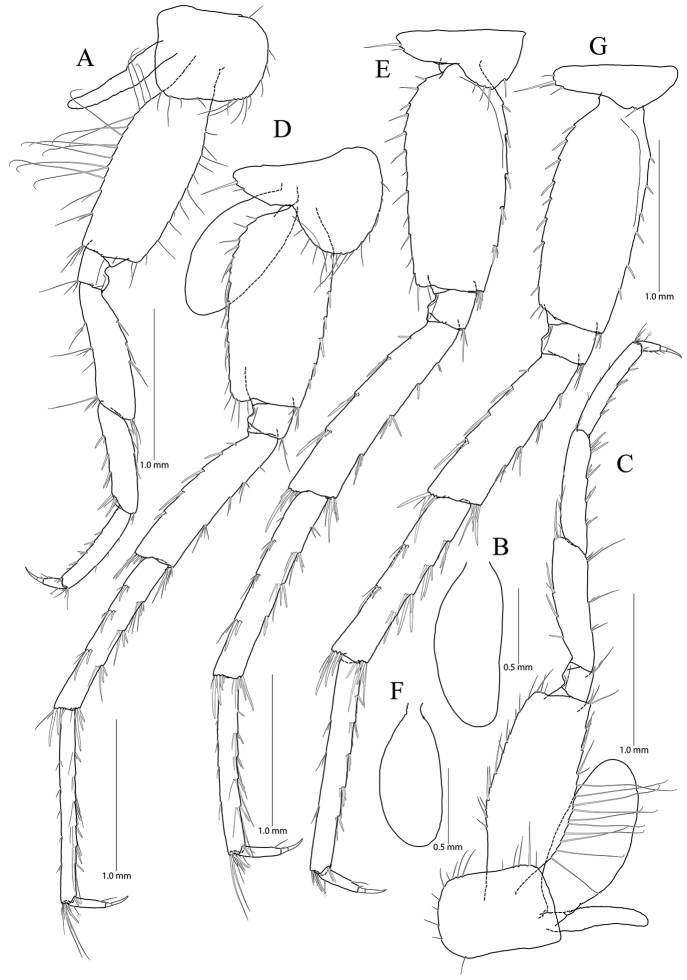
*Pseudocrangonyx gracilipes* sp. nov. Holotype: female, NIBRV0000862808, 9.9 mm, from Gosugul Cave, South Korea. **A** pereopod 3 **B** coxal gill of pereopod 3 **C** pereopod 4 **D** pereopod 5 **E** pereopod 6 **F** coxal gill of pereopod 6 **G** pereopod 7.

Pereopod 3 (Fig. 7A, B) coxa subrectangular, 1.27 × wider than long, anteriorly convex, with ten setae anteriorly, ventral margin a little concave, with four setae posteriorly; coxal gill present, subovate; oostegite present, narrow, shorter than that of coxal gill, without marginal setae; basis expanded, 0.70 × as wide and 2.55 × as long as coxa, width longest at proximal 1/3 and slightly diminished distally, anterior margin lined with simple setae, posterior margin lined with elongate setae; ischium 0.16 × as long as basis; merus anterodistally expanded, 0.30 × wider than long, 0.60 × as long as basis, anterodistal corner produced, apex blunt; carpus not expanded, 0.48 × as long as basis; propodus linear, 0.93 × as long as carpus; dactylus 0.44 × as long as propodus, unguis developed.

Pereopod 4 (Fig. 7C) similar to pereopod 3 except that carpus and propodus 1.11 × as long as those of pereopod 3; different number or position of several setae.

Pereopod 5 (Fig. 7D) coxa bilobate, anterior lobe larger than posterior lobe, expanded ventrally (1.14 × longer than wide), margin rounded, lined with 13 simple setae; posterior lobe with two setae at posterior corner (ventral one short); coxal gill present, subovate; oostegite present, narrow, shorter than that of coxal gill, length shortest, without marginal setae; basis expanded, subrectangular, 0.46 × wider than long, anterior margin slightly convex, lined with five single robust setae and one pair of robust setae, posterior margin lined with 18 simple setae, distal corner produced and forming angle; merus posterodistally expanded, 0.40 × as wide and 0.86 × as long as basis, anterior margin with one simple seta and three clusters of simple setae marginally, and one cluster of setae distally, posterior margin with five single robust setae marginally, and one cluster of robust and simple setae distally; carpus not expanded 1.00 × as long as basis, anterior and posterior margins with three marginal clusters and one distal cluster of setae, respectively; propodus linear, 1.11 × as long as carpus, anterior margin with six setal clusters (longest seta of distal cluster not exceeding end of propodus) and one pair of locking robust setae distally, posterior margin with five pairs of short setae marginally and one cluster of elongate setae distally; dactylus slender, 0.32 × as long as propodus, unguis developed.

Pereopod 6 (Fig. 7E, F) 1.12 × as long as pereopod 5; coxa bilobate, anterior lobe 0.60 × as long as that of pereopod 5, with four setae on ventral margin, posterior lobe expanded backward, with two setae at posterior corner (ventral one very short); coxal gill present, subovate; oostegite absent; basis expanded, subrectangular, 1.13 × as long and 1.08 × as wide as that of pereopod 5, 0.46 × wider than long, anterior margin slightly convex, lined with three single robust setae and two clusters of robust setae, with one cluster of robust setae at distal corner, posterior margin lined with 14 setae, posterodistal corner produced forming an angle; merus posterodistally expanded, 1.24 × as long as that of pereopod 5, 0.44 × as wide and 0.95 × as long as basis, anterior and posterior margins with four single setae or clusters of setae marginally and one cluster of setae distally, respectively; carpus not expanded, 0.73 × as long as basis, anterior margin with four clusters of setae marginally and one cluster of setae distally, posterior margin with one simple seta and three clusters of simple and robust setae marginally, and one cluster of setae distally; propodus linear, 0.88 × as long as carpus, anterior margin with one robust seta and four clusters of setae marginally (longest seta of distal cluster not exceeding end of propodus) and one pair of locking robust setae distally, posterior margin with four pairs of short setae marginally and one cluster of elongate setae distally; dactylus 0.39 × as long as propodus, unguis developed.

Pereopod 7 (Fig. 7G) 0.97 × as long as pereopod 6; coxa unilobed, subtriangular, 0.73 × as long as that of pereopod 6, with one seta on ventral margin, posteriorly expanded with four setae at posterior corner; coxal and oostegite absent; basis expanded, subrectangular, 1.01 × as long and 0.87 × as wide as that of pereopod 6, 0.39 × wider than long, anterior margin slightly convex, lined with six robust setae, with one cluster of setae at distal corner, posterior margin lined with eleven setae, distal corner slightly produced but angle smaller than in pereopods 6 and 7; merus posterodistally expanded, 0.82 × as long as that of pereopod 6, 0.57 × as wide and 0.77 × as long as basis, anterior margin with four marginal clusters and one distal cluster of setae, posterior margin with one single robust seta and three clusters of simple and robust setae marginally, and one cluster of simple and robust setae distally; carpus not expanded, rectangular, 1.11 × as long as merus, anterior margin with four clusters of setae marginally and one cluster of setae distally, posterior margin with two clusters of setae marginally and one cluster of setae distally; propodus linear, 1.22 × as long as carpus, anterior margin with five clusters of setae (without elongate setae) and one pair of locking robust setae distally; posterior margin with three pairs of short setae marginally and one cluster of setae distally (shorter than those of pereopods 6 and 7); dactylus 0.36 × as long as propodus, unguis developed.

**Figure 8.**
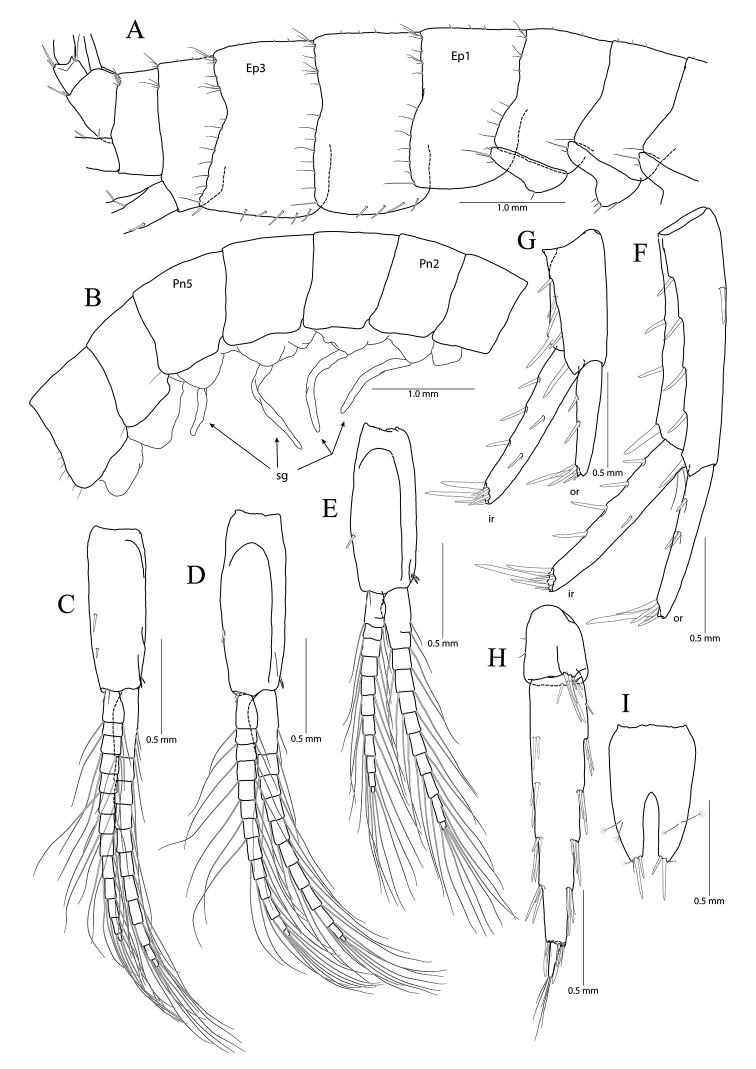
*Pseudocrangonyx gracilipes* sp. nov. Holotype: female, NIBRV0000862808, 9.9 mm, from Gosugul Cave, South Korea. **A** epimeral plates 1–3 **B** sternal gills **C** pleopod 1 **D** pleopod 2 **E** pleopod 3 **F** uropod 1 **G** uropod 2 **H** uropod 3 **I** telson. Abbreviations: Ep, epimeral plate; Pn, pereonite; sg, sternal gill; ir, inner ramus; and or, outer ramus.

Sternal gills (Fig. 8B) present from pereonites 2–5 (1+1+1+1 in formulae), narrower than oostegites, shortest in that of pereonite 5.

Epimeral plate 1 subquadrate, slightly produced posteroventrally, ventral margin without setae, posterior margin with six setae, posterodistal corner slightly notched bearing one elongate seta. Epimeral plate 2 subquadrate, larger than epimeron 1, ventral margin with four submarginal setae anteriorly, posterior margin with seven setae, posterodistal corner slightly notched bearing one elongate setae. Epimeral plate 3 posterior margin with eight setae, posterodistal corner produced backward, with weak notch, bearing one elongate seta, ventral margin with five submarginal setae anteriorly, not emarginated (Fig. 8A).

Pleopod 1 (Fig. 8C) peduncle with one pair of retinaculae mediodistally and one pair of simple setae laterodistally, with two robust setae laterally; outer ramus 1.55 × as long as peduncle, composed of 13 articles; inner ramus 1.75 × as long as peduncle, composed of eleven articles (coalesced 1^st^ article as long as proximal three articles of outer ramus combined).

Pleopod 2 (Fig. 8D) peduncle 1.11 × as long as that of pleopod 1, with one pair of retinaculae mediodistally and one pair of simple setae laterodistally, with one robust seta laterally; outer ramus 1.42 × as long as peduncle, composed of 13 articles; inner ramus 1.48 × as long as peduncle, composed of eleven articles (coalesced 1^st^ article 0.90 × as long as proximal three articles of outer ramus combined).

Pleopod 3 (Fig. 8E) 0.91 × as long as pleopod 2; peduncle 0.92 × as long as that of pleopod 2, with one pair of retinaculae mediodistally and one pair of simple setae laterodistally, with one robust seta laterally; outer ramus 1.20 × as long as peduncle, composed of eleven articles, 1^st^ article not fully coalesced and with trace; inner ramus 1.46 × as long as outer ramus, composed of ten articles, 1^st^ article not fully coalesced and with trace, 9^th^ and 10^th^ articles coalesced.

Uropod 1 (Fig. 8F) peduncle with one basofacial seta, with four marginal setae and one distal seta dorsolaterally, with two marginal setae and one distal seta dorsomedially; outer ramus 0.63 × as long as peduncle, with one robust seta dorsomedially and two robust setae dorsolaterally, apical cluster composed of five robust setae; inner ramus 1.15 × as long as outer ramus, with one robust seta dorsolaterally and four robust setae dorsomedially, apical cluster composed of five robust setae and one simple and one penicillate seta, with two elongate setae subproximally on ventral margin.

Uropod 2 (Fig. 8G) 0.59 × as long as uropod 1; peduncle 0.52 × as long as that of uropod 1, with three marginal setae and one distal seta dorsolaterally, with one marginal seta and one distal seta dorsomedially; outer ramus 0.84 × as long as peduncle, with two robust setae dorsolaterally, apical cluster composed of five robust setae; inner ramus 1.50 × as long as outer ramus, with two robust setae dorsolaterally and three robust setae dorsomedially, apical cluster composed of seven robust setae and one penicillate seta.

Uropod 3 (Fig. 8H) uniramous, 0.83 × as long as uropod 1; peduncle short, 0.55 × as long as uropod 2, with two minute setae on medial margin, with one dorsal and one ventral cluster of robust setae laterodistally; ramus 4.17 × as long as peduncle, bi-articulate, proximal article gradually diminished distally, with five lateral and five medial clusters of setae (longest seta of each distal cluster exceeding end of last article), distal article 0.13 × as long as proximal article, with five simple setae apically.

Telson (Fig. 8I) 0.63 × as wide as long, cleft for 47% of length, each lobe with one pair of penicillate setae dorsally, and one penicillate seta and two robust setae on apex.

###### Remarks.

*Pseudocrangonyx gracilipes* sp. nov. resembles with its several congeners including *P. bohaensis* (Derzhavin, 1927), *P. yezonis* Akatsuka & Komai, 1992, *P. relicta* Labay, 1999, and *P. camtschaticus* Birstein, 1955 in having the notched posterodistal corners of epimeral plates 2 and 3. However, this new species is distinguished from these species by the more produced posterodistal corner of epimeral plate 3 (Birstein 1955, Akatsuka and Komai 1922, Labay 1999).

This character state of the epimeral plates is also observed in *P. villosus* sp. nov., but *P. gracilipes* sp. nov. has four plumose setae on the inner plate of maxilla 1 (compared to seven in *P. villosus* sp. nov.), four plumose setae on inner plate of maxilla 2 (compared to ten in *P. villosus* sp. nov.), both three rastellate setae on gnathopods 1 and 2 (compared to two rastellate setae in *P. villosus* sp. nov.), and the presence of the more slender and elongated pereopods 5–7.

##### Pseudocrangonyx crassus sp. nov.

Taxon classificationAnimaliaAmphipodaPseudocrangonyctidae

99044872-F6DE-5F31-AE15-DD3F2FAFE677

http://zoobank.org/BE3227BA-231F-4FA5-B280-B814B7C4C95E

**Figure 9.**
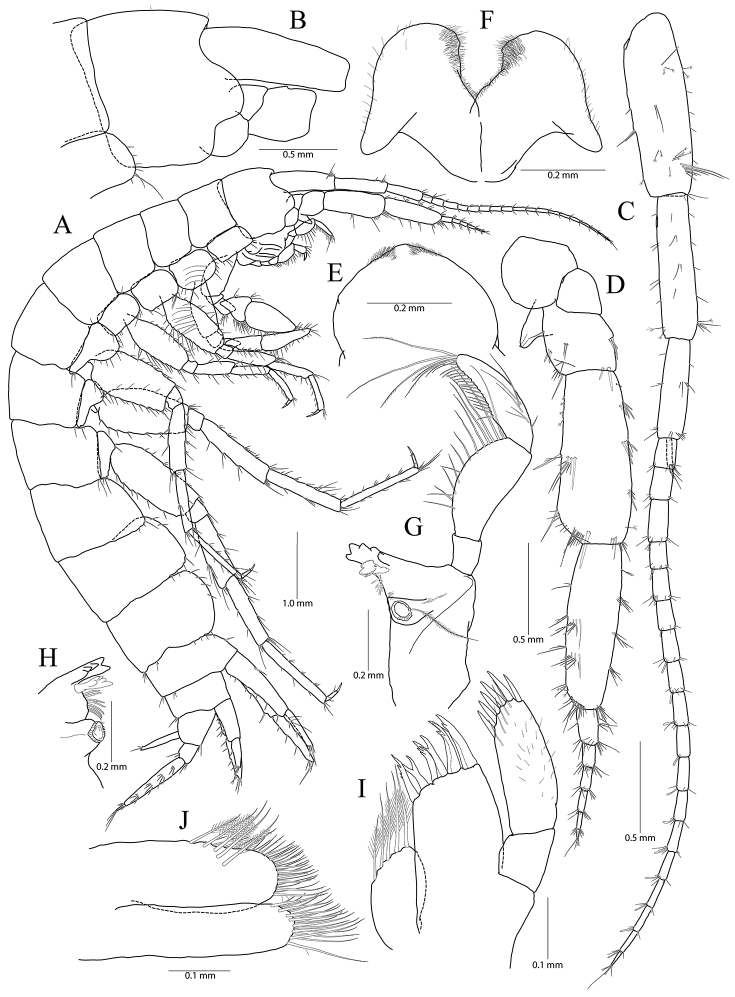
*Pseudocrangonyx crassus* sp. nov. Holotype: male, NIBRV0000862809, 10.6 mm, from Gosugul Cave, South Korea. **A** habitus **B** head **C** antenna 1 **D** antenna 2 **E** upper lip **F** lower lip **G** right mandible **H** left mandible **I** maxilla 1 **J** maxilla 2.

**Figure 10.**
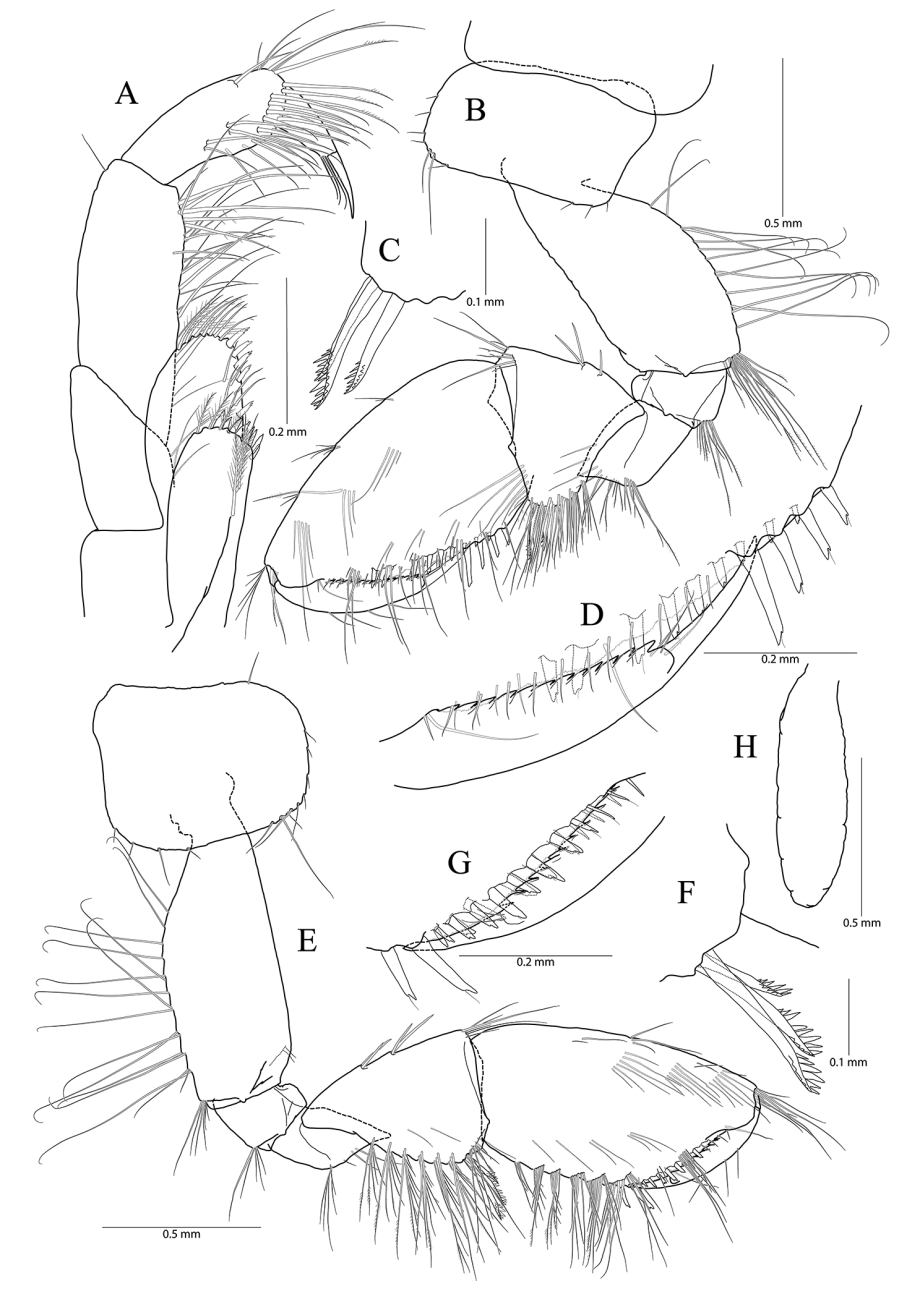
*Pseudocrangonyx crassus* sp. nov. Holotype: male, NIBRV0000862809, 10.6 mm, from Gossigul Cave, South Korea. **A** maxilliped **B** gnathopod 1 **C** rastellate setae gnathopod 1 **D** and palm of gnathopod 1 **E** gnathopod 2 **F** rastellate setae of gnathopod 2 **G** palm of gnathopod 2 **H** coxal gill of gnathopod 2.

**Figure 11.**
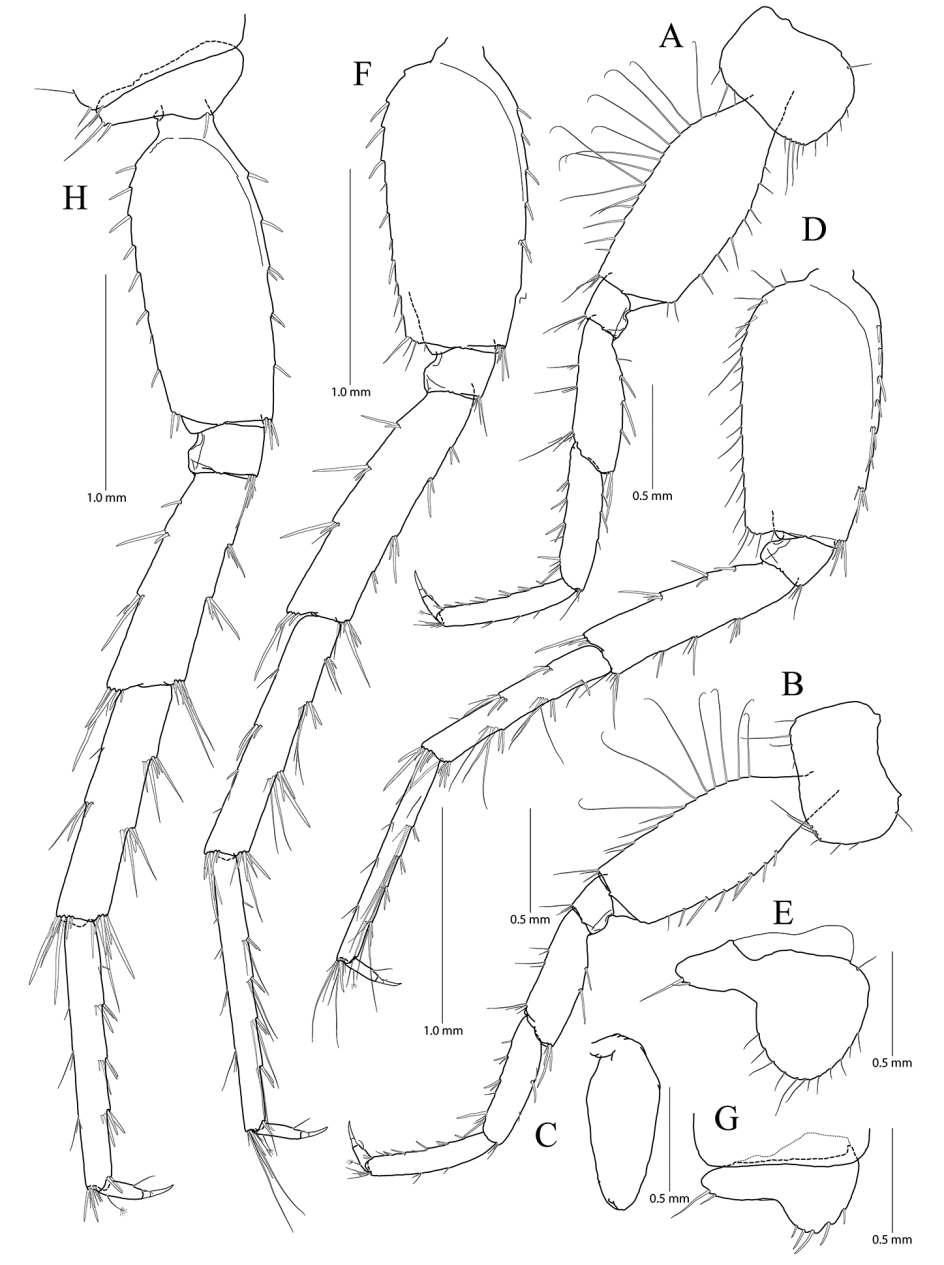
*Pseudocrangonyx crassus* sp. nov. Holotype: male, NIBRV0000862809, 10.6 mm, from Gossigul Cave, South Korea. **A** pereopod 3 **B** pereopod 4 **C** coxal gill of pereopod 4 **D** pereopod 5 **E** coxa of pereopod 5 **F** pereopod 6 **G** coxal gill of pereopod 7 **H** pereopod 7.

**Figure 12.**
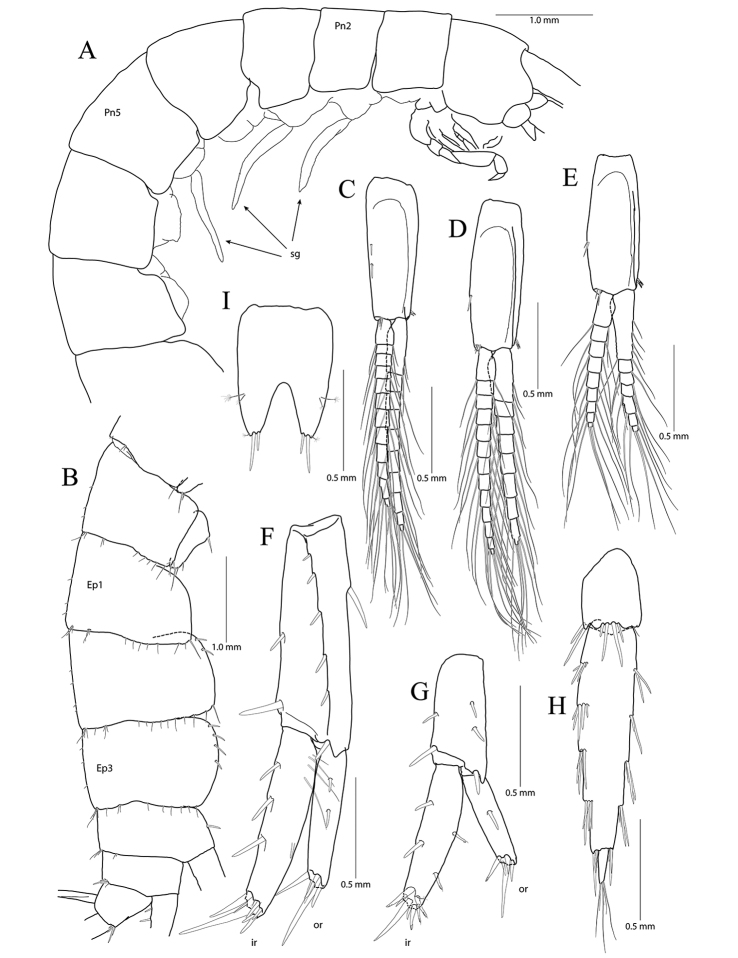
*Pseudocrangonyx crassus* sp. nov. Holotype: male, NIBRV0000862809, 10.6 mm, from Gossigul Cave, South Korea. **A** sternal gills **B** epimeral plates 1–3 **C** pleopod 1 **D** pleopod 2 **E** pleopod 3 **F** uropod 1 **G** uropod 2 **H** uropod 3 **I** telson. Abbreviations: Ep, epimeral plate; Pn, pereonite; sg, sternal gill; ir, inner ramus; and or, outer ramus.

Figs 9 , 10 , 11 , 12

###### Korean name.

Keun-deo-deum-i-dong-gul-yeop-sae-u, new

###### Type locality.

Gossigul Cave, Yeongwol-gun, Gangwon-do, South Korea; 37°08'00"N, 128°31'21"E.

**Material examined**. Type material. ***Holotype***: 1 male, 10.6 mm, NIBRIV0000862809, collected from the type locality on 19 Jan 2001 by YG Choi.

###### Etymology.

The specific name originates from the Latin word *crassus* meaning thick. This name refers to the shape of the 4^th^ and 5^th^ articles of antenna 2.

###### Diagnosis.

Antenna 2, 4^th^ and 5^th^ peduncular articles expanded, plump medially, calceoli and aesthetascs absent. Maxilla 1 inner lobe with five plumose setae on apical margin; 2^nd^ palp article with six robust setae along distomedial to apical margins. Maxilla 2 inner lobe with one oblique row of five plumose setae on surface; outer lobe without plumose setae. Gnathopods 1 and 2 each possessing carpus with three rastellate setae, respectively. Pereopods 3 and 4 basis bearing elongate setae on posterior margins. Sternal gills present on pereonites 2, 3, and 5 (1+1+0+1 in formulae). Epimeral plate 3 posteroventral corner slightly produced. Telson cleft for 39% of length.

###### Description.

Holotype male: Body (Fig. 9A) approximately 10.6 mm long.

Head (Fig. 9B) 1.30 × as long as pereonite 1; rostrum reduced, with one minute seta; lateral cephalic lobe anteriorly expanded, apex rounded, slightly dilated anteroventrally; antennal sinus not deep; eye absent.

Antenna 1 (Fig. 9C) 0.50 × as long as body; 1^st^–3^rd^ peduncular articles length ratio of 1.00 : 0.80 : 0.56; 1^st^ article stouter than 2^nd^ and 3^rd^ articles, without robust setae on posterior margin; accessory flagellum bi-articulate, last article very reduced; flagellum 1.28 × as long as peduncles, composed of 18 articles, calceoli absent, aesthetascs present from proximal 3^rd^ to last articles.

Antenna 2 (Fig. 9D) 0.62 × as long as antenna 1; antennal cone developed, apex rounded; 4^th^ and 5^th^ peduncular articles expanded, plump medially; 4^th^ article 0.93 × as long as 1^st^ peduncular article of antenna 1, anterior margin convex, with three clusters of robust setae, posterior margin distally expanded, rounded posterodistally; 5^th^ article expanded at the middle, 0.98 × as long and 0.69 × as wide as 4^th^ article, calceoli absent; flagellum composed of six articles, 0.77 × as long as 5^th^ peduncular article; calceoli and aesthetascs absent.

Upper lip (Fig. 9E) anteriorly rounded, apex covered with minute setae, not notched.

Lower lip (Fig. 9F) inner lobes indistinct; outer lobes covered with minute setae; mandibular processes developed.

Mandible (Fig. 9G, H) incisor 5-dentate on both sides; lacinia mobilis tri-cuspidate (two finely dentate and one not dentate) on right and bi-furcate (one 4-dentate and the other not dentate) on left side; seven and eight raker setae present on right and left sides; molar process columnar, triturative, with one plumose seta on right side only; palp tri-articulate; 2^nd^ article convex and with 14 setae medially; 3^rd^ article subfalcate, 1.2 × as long as 2^nd^ article, medial margin lined with 23 setae from medial margin to apex.

Maxilla 1 (Fig. 9I) inner lobe subrhomboid, with five plumose setae on apical margin; outer lobe with seven dentate robust setae; palp bi-articulate, 2^nd^ article apex slightly exceeding apical setae of outer plate, with six robust setae along distomedial to apical margins, with one oblique row of five setae subdistally.

Maxilla 2 (Fig. 9J) inner lobe slightly shorter but wider than outer lobe, with one oblique row of five plumose setae on surface and two rows of simple setae on apical margin; outer lobe apical margin also with two rows of simple setae (without plumose setae).

Maxilliped (Fig. 10A) inner lobe subrectangular, with eight dentate robust setae on apex and eight plumose setae mediodistally and subapically; outer lobe elongate semicircular, 0.70 × as long as 2^nd^ palp article, with five dentate robust setae and seven weakly plumose setae apically; palp composed of four articles; 2^nd^ article with many setae on medial margin; 3^rd^ article slightly dilated distally, 0.70 × as long as 2^nd^ article; 4^th^ article falcate, 0.49 × as long as 3^rd^ article, apical setae 0.76 × as long as 4^th^ article.

Gnathopod 1 (Fig. 10B-D) coxa subrectangular, 1.63 × wider than long, anteroventral corner not produced, rounded, with seven setae marginally, ventral margin slightly concave, with three setae posteriorly, posterior margin humped proximally; basis obtuse trapezoidal, posteriorly expanded, 0.49 × wider than long, lined with elongate simple setae posteriorly, anterior margin without setae; ischium 0.28 × as long as basis, with small anterior lobe; carpus 0.62 × as long as basis, with one single seta and one pair of robust setae on anterior margin, carpal lobe not developed, apex rounded with three rastellate setae and many simple or serrate setae; propodus subovate, 1.24 × as long and 1.39 × as wide as basis, posterior margin short, 0.24 × as long as anterior margin, with four robust setae laterally along posterior margin distally and nine robust setae medially along posterior margin and palm (all medial setae small, but lateral setae larger and successively increasing distally), palm irregular, finely serrated, defined by largest lateral seta of posterior margin; dactylus as long as palm, apex reaching largest lateral seta of posterior margin, inner margin toothed, outer margin with four setae, unguis developed.

Gnathopod 2 (Fig. 10E-H) 1.07 × as long as gnathopod 1; coxa 1.29 × as long as that of gnathopod 1, 1.29 × wider than long, anterior and ventral margins not produced, rounded, lined with eight setae, ventral margin with four setae posteriorly, posterior margin humped proximally; coxal gill present, subovate; basis subtrapezoidal, posteriorly expanded, 0.92 × as wide as that of gnathopod 1, 0.36 × wider than long, lined with elongate simple setae posteriorly, anterior margin with two setae subdistally; ischium 0.23 × as long as basis, with small anterior lobe; carpus 0.60 × as long as basis, with two pairs of robust setae on anterior margin, carpal lobe not developed, broad, with seven clusters of many simple or serrate setae, with three rastellate setae distally; propodus trapezoidal, 0.93 × as long and 1.31 × as wide as basis, anterior margin slightly convex, posterior margin 0.52 × as long as anterior margin, lined with six clusters of elongate setae, palm irregular, finely serrated, lined with six medial and nine lateral robust setae (palm defined by two unequal setae distally among lateral setae); dactylus as long as palm, inner margin toothed, outer margin with three setae, unguis developed.

Pereopod 3 (Fig. 11A) coxa rectangular, 1.35 × wider than long, with ten setae anteriorly and three setae posteroventrally, ventral margin a little concave; coxal gill present, subovate; basis expanded, 0.78 × as wide and 2.65 × as long as coxa, width longest at proximal 1/3 and slightly diminished distally, anterior margin slightly convex, lined with nine simple setae, posterior margin lined with 18 elongate setae; ischium 0.16 × as long as basis; merus anterodistally expanded, 0.36 × wider than long, 0.51 × as long as basis, anterodistal corner produced, apex blunt; carpus not expanded, 1.00 × as long as merus; propodus linear, 1.06 × as long as carpus; dactylus 0.43 × as long as propodus, unguis developed.

Pereopod 4 (Fig. 11B, C) each article similar to but slightly shorter than those of pereopod 3, except for the number and positions of several setae.

Pereopod 5 (Fig. 11D, E) coxa with additional tumid lobe proximally, bilobate, anterior lobe larger than posterior lobe, expanded ventrally (1.09 × longer than wide), margin rounded, lined with twelve simple setae; posterior lobe with two setae at posterior corner (ventral one very short); coxal gill present, subovate; basis expanded, subrectangular, 0.52 × wider than long, anterior margin slightly convex, lined with five single robust setae and two clusters of robust setae, with one cluster of elongate setae at distal corner, posterior margin lined with 21 simple setae, with two submarginal robust setae proximally, distal corner produced forming an angle; merus posterodistally expanded, 0.38 × as wide and 0.78 × as long as basis, anterior margin with three clusters of setae marginally and one cluster of setae distally, posterior margin with one single robust seta and two clusters of setae marginally, and one cluster of setae distally; carpus not expanded, 0.92 × as long as merus, anterior margin with two clusters of setae and one single seta marginally, and one cluster of setae distally, posterior margin with three clusters bearing elongate simple setae marginally and one cluster of setae distally; propodus linear, 1.17 × as long as carpus, anterior margin with six setal clusters (all clusters with elongate setae and those of distal cluster exceeding end of propodus) and one pair of locking robust setae distally, posterior margin with three marginal clusters of short setae and one cluster of elongate setae distally; dactylus slender, 0.29 × as long as propodus, unguis developed.

Pereopod 6 (Fig. 11F, G) 1.21 × as long as pereopod 5; coxa with additional tumid lobe proximally, bilobate, anterior lobe 0.80 × as long as that of pereopod 5, with five robust setae on ventral margin, posterior lobe expanded backward, with two setae at posterior corner (ventral one short); coxal gill present, subovate; basis expanded, subrectangular, 1.12 × as long and 1.02 × as wide as that of pereopod 5, 0.47 × wider than long, anterior margin slightly convex, lined with more than four single robust setae and one clusters of robust setae, with one cluster of setae at distal corner, posterior margin lined with 14 setae, distal corner produced forming an angle; merus posterodistally expanded, 1.28 × as long as that of pereopod 5, 0.41 × as wide and 0.89 × as long as basis, anterior margin with four clusters of setae marginally and one cluster of setae distally, posterior margin with three clusters of setae marginally and one cluster of setae distally; carpus not expanded, 0.85 × as long as basis, anterior margin with three clusters of setae marginally and one cluster of simple and robust setae distally, posterior margin with one simple seta and three clusters of simple or robust setae marginally, and one cluster of setae distally; propodus linear, 1.08 × as long as carpus, anterior margin with one single seta and four clusters of setae marginally (longest seta of distal cluster exceeding end of propodus) and one pair of locking robust setae distally, posterior margin with one single seta, four pairs of short setae marginally and one distal cluster of elongate setae distally; dactylus 0.28 × as long as propodus, unguis developed.

Pereopod 7 (Fig. 11H) 0.98 × as long as pereopod 6; coxa unilobed, subtriangular, 0.65 × as long as that of pereopod 6, with one seta on ventral margin, posteriorly expanded with three setae at posterior corner; basis expanded, subrectangular, 1.17 × as long and 0.92 × as wide as that of pereopod 6, 0.41 × wider than long, anterior margin slightly convex, lined with three single robust setae and two clusters of robust setae, with one cluster of setae at distal corner, posterior margin lined with ten setae, distal corner slightly produced but angle smaller than those of pereopods 6 and 7; merus posterodistally expanded, 0.82 × as long as that of pereopod 6, 0.55 × as wide and 0.68 × as long as basis, anterior margin with two clusters of setae marginally and one cluster of setae distally, posterior margin with one single seta and two clusters of setae marginally and one cluster of setae distally; carpus not expanded, rectangular, 0.22 × as wide as long, 1.13 × as long as merus, anterior margin with two marginal clusters and one distal cluster of simple and robust setae, posterior margin with two clusters of setae marginally and one cluster of simple and robust setae distally; propodus linear, 1.10 × as long as carpus, anterior margin with four setal clusters (without elongate setae) marginally and one pair of locking robust setae distally, posterior margin with one single seta and two pairs of short setae marginally, and one cluster of setae distally (shorter than those of pereopods 6 and 7), dactylus 0.29 × as long as propodus, unguis developed.

Sternal gills (Fig. 12A) present on pereonites 2, 3, and 5 (1+1+0+1 in formulae), narrow, not short in pereonite 5.

Epimeral plate 1 subquadrate, slightly produced posteroventrally, ventral margin without setae, posterior margin with seven setae, posterodistal corner slightly notched bearing one elongate seta. Epimeral plate 2 subquadrate, larger than epimeron 1, ventral margin with two submarginal setae anteriorly, posterior margin with nine setae, posterodistal corner slightly notched bearing one elongate seta. Epimeral plate 3 posterior margin with six setae, posterodistal corner a little produced and without notch, ventral margin with four submarginal setae anteriorly, not emarginated (Fig. 12B).

Pleopod 1 (Fig. 12C) peduncle with one pair of retinaculae mediodistally and three simple setae laterodistally, with two robust setae laterally; outer ramus 1.40 × as long as peduncle, composed of twelve articles, 11^th^ and 12^th^ articles coalesced; inner ramus 1.54 × as long as peduncle, composed of ten articles (1^st^ coalesced article exceeding three proximal articles of outer ramus combined).

Pleopod 2 (Fig. 12D) peduncle 1.06 × as long as that of pleopod 1, with one pair of retinaculae mediodistally and one cluster of three simple setae laterodistally, with one robust seta laterally; outer ramus 1.43 × as long as peduncle, composed of 13 articles; inner ramus 0.92 × as long as outer ramus, composed of eight articles (1^st^ coalesced article exceeding two proximal articles of outer ramus combined, last articles coalesced).

Pleopod 3 (Fig. 12E) 0.78 × as long as pleopod 2; peduncle 0.95 × as long as that of pleopod 2, with one pair of retinaculae mediodistally and one pair of simple setae laterodistally, with one pair of setae laterally; outer ramus 1.00 × as long as peduncle, composed of nine articles, 1^st^ article not fully coalesced and with trace; inner ramus 1.06 × as long as outer ramus, composed of seven articles, 1^st^ article not fully coalesced and with trace, 5^th^ article coalesced.

Uropod 1 (Fig. 12F) peduncle with one basofacial seta, with five marginal setae and one distal seta dorsolaterally, with one marginal seta and one distal seta dorsomedially; outer ramus 0.60 × as long as peduncle, with two robust setae dorsolaterally, apical cluster composed of five robust setae; inner ramus 1.31 × as long as outer ramus, with one robust seta dorsolaterally and three robust setae dorsomedially, apical cluster composed of seven robust setae and one penicillate seta, with four elongate setae on ventral margin subproximally.

Uropod 2 (Fig. 12G) 0.63 × as long as uropod 1; peduncle 0.55 × as long as that of uropod 1, with two marginal setae and one distal seta dorsolaterally, with one distal seta and one marginal seta dorsomedially; outer ramus 0.76 × as long as peduncle, with one robust seta dorsolaterally, apical cluster composed of five robust setae (one of them with abnormal apex); inner ramus 1.52 × as long as outer ramus, with two robust setae (bearing abnormal apices) dorsolaterally and two robust setae dorsomedially, apical cluster composed of seven robust setae (three of them with abnormal apices) and one penicillate seta.

Uropod 3 (Fig. 12H) uniramous, 0.85 × as long as uropod 1; peduncle short, 0.61 × as long as uropod 2, distal margin with one dorsal and one medial clusters of robust setae; ramus 3.25 × as long as peduncle, bi-articulate, proximal article gradually diminished in width, with five lateral and five medial clusters of setae (longest seta of each distal cluster exceeding end of last article), distal article 0.16 × as long as proximal article, with six simple setae apically.

Telson (Fig. 12I) 0.73 × as wide as long, cleft for 39% of length, each lobe with one pair of penicillate setae dorsally, and one penicillate seta and two robust setae on apex.

###### Remarks.

*Pseudocrangonyx crassus* sp. nov. is very similar to *P. tiunovi* Sidorov & Gontcharov, 2013 in having expanded peduncular articles and a reduced flagellum of antenna 2, in addition to bearing similarities in the general shape and length ratio of the articles in the gnathopods and pereopods. However, this new species differs from *P. tiunovi* by possessing more expanded 4^th^ and 5^th^ peduncular articles of antenna 2, longer palp articles of maxilla 1 bearing more robust setae (six in *P. crassus* sp. nov. compared to four in *P. tiunovi*), a longer 3^rd^ palp article on the maxilliped compared to *P. tiunovi*, three rastellate setae in both gnathopods 1 and 2 (one and two on gnathopods 1 and 2, respectively, in *P. tiunovi*), more elongate setae on the basis posterior margins on pereopods 3 and 4, a more expanded anterior lobe of coxa 5, a slightly produced posteroventral corner of epimeral plate 3 (more produced in *P. tiunovi*), and a telson cleft for 39% of length (cleft for 20% of length in *P. tiunovi*) (Sidorov and Gontcharov 2013).

##### Pseudocrangonyx minutus sp. nov.

Taxon classificationAnimaliaAmphipodaPseudocrangonyctidae

D7D736DD-6CD6-5BF8-BF24-AC6586B4AAB9

http://zoobank.org/697E91AE-9C90-4F6C-BEB4-F3CC80DA777A

**Figure 13.**
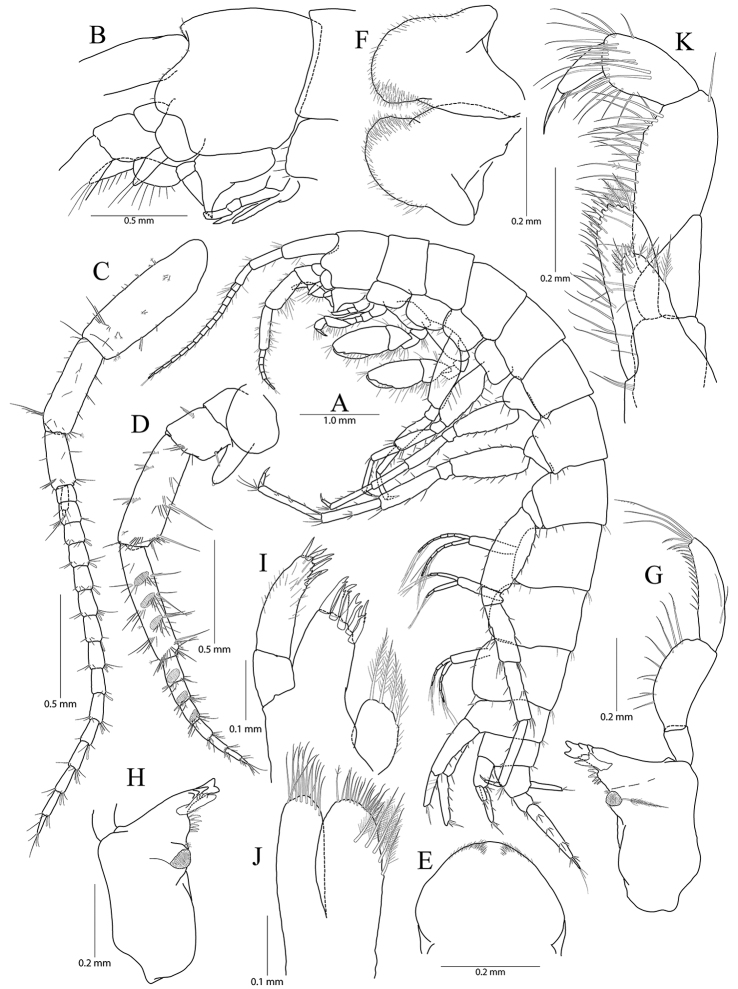
*Pseudocrangonyx minutus* sp. nov. Holotype: female, NIBRV0000862810, 9.1 mm, from Gageo Is., South Korea. **A** habitus **B** head **C** antenna 1 **D** antenna 2 **E** upper lip **F** lower lip **G** right mandible **H** left mandible **I** maxilla 1 **J** maxilla 2 **K** maxilliped.

**Figure 14.**
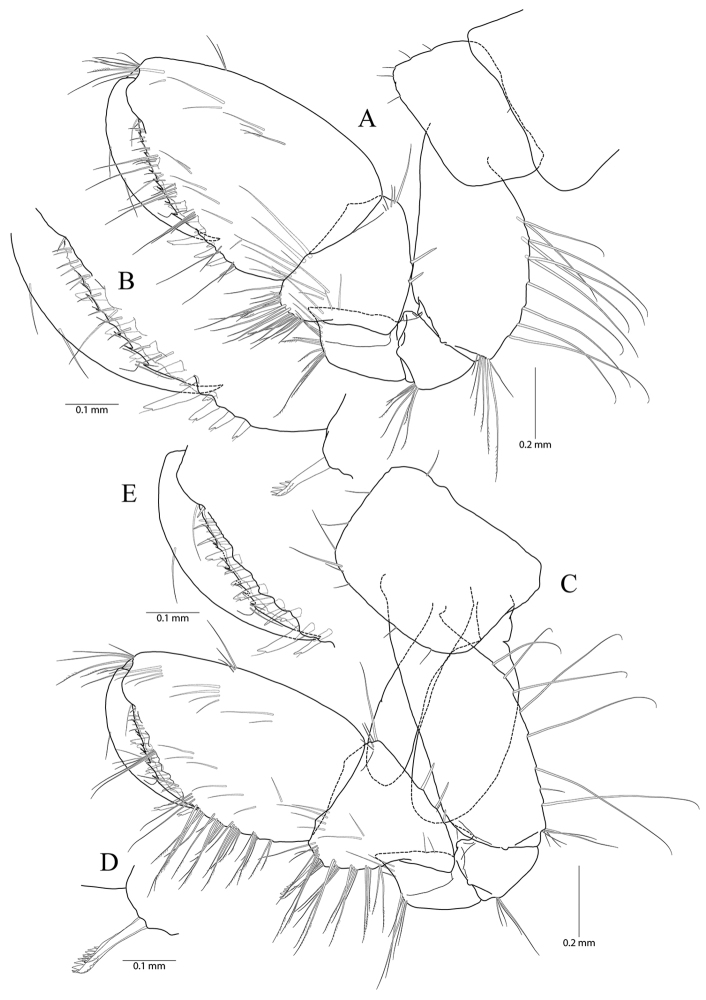
*Pseudocrangonyx minutus* sp. nov. Holotype: female, NIBRV0000862810, 9.1 mm, from Gageo Is., South Korea. **A** gnathopod 1 **B** rastellate setae and palm of gnathopod 1 **C** gnathopod 2 **D** rastellate setae of gnathopod 2 **E** palm of gnathopod 2.

**Figure 15.**
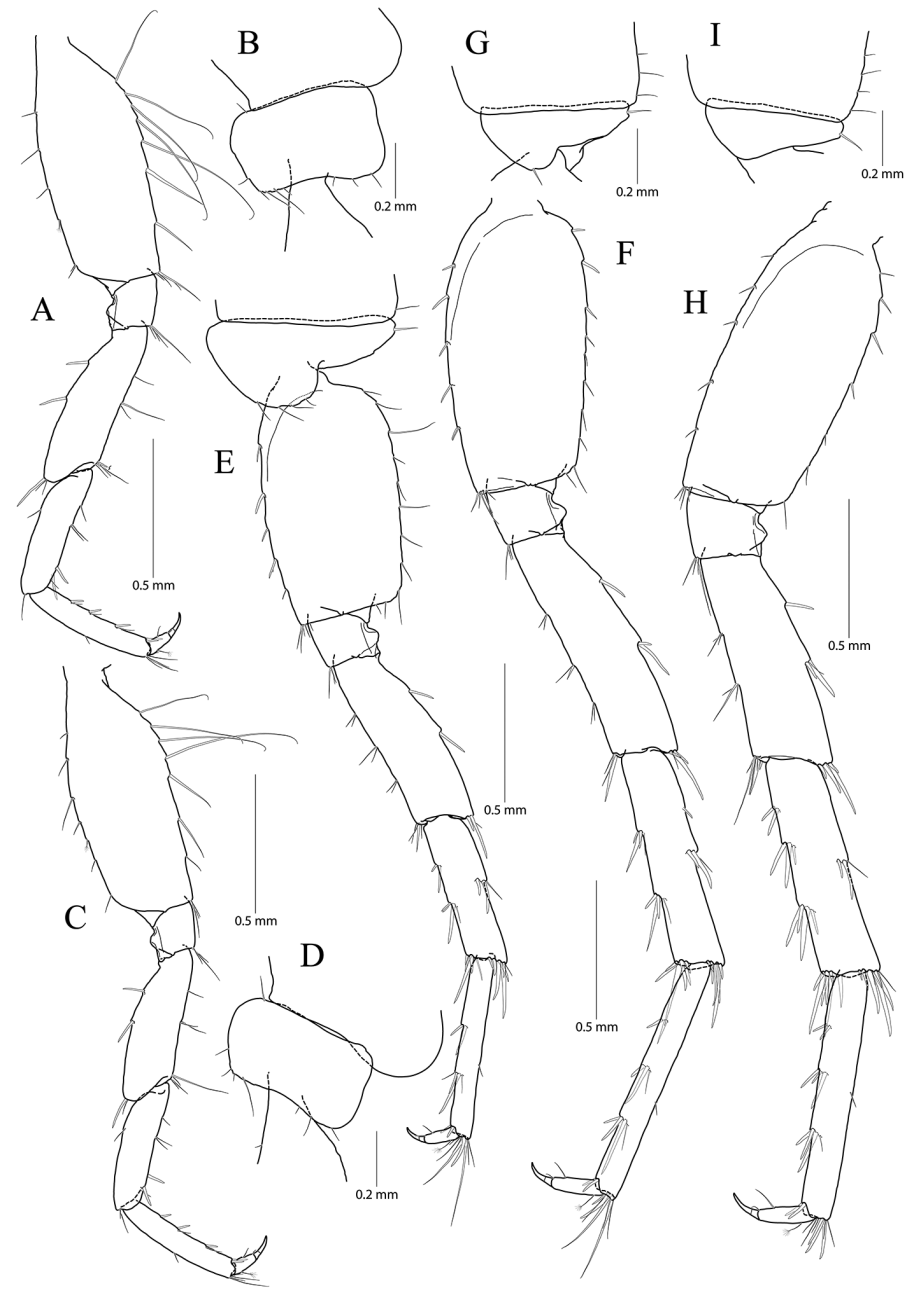
*Pseudocrangonyx minutus* sp. nov. Holotype: female, NIBRV0000862810, 9.1 mm, from Gageo Is., South Korea. **A** pereopod 3 **B** coxal gill of pereopod 3 **C** pereopod 4 **D** coxal gill of pereopod 4 **E** pereopod 5 **F** pereopod 6 **G** coxal gill of pereopod 6 **H** pereopod 7 **I** coxal gill of pereopod 7.

**Figure 16.**
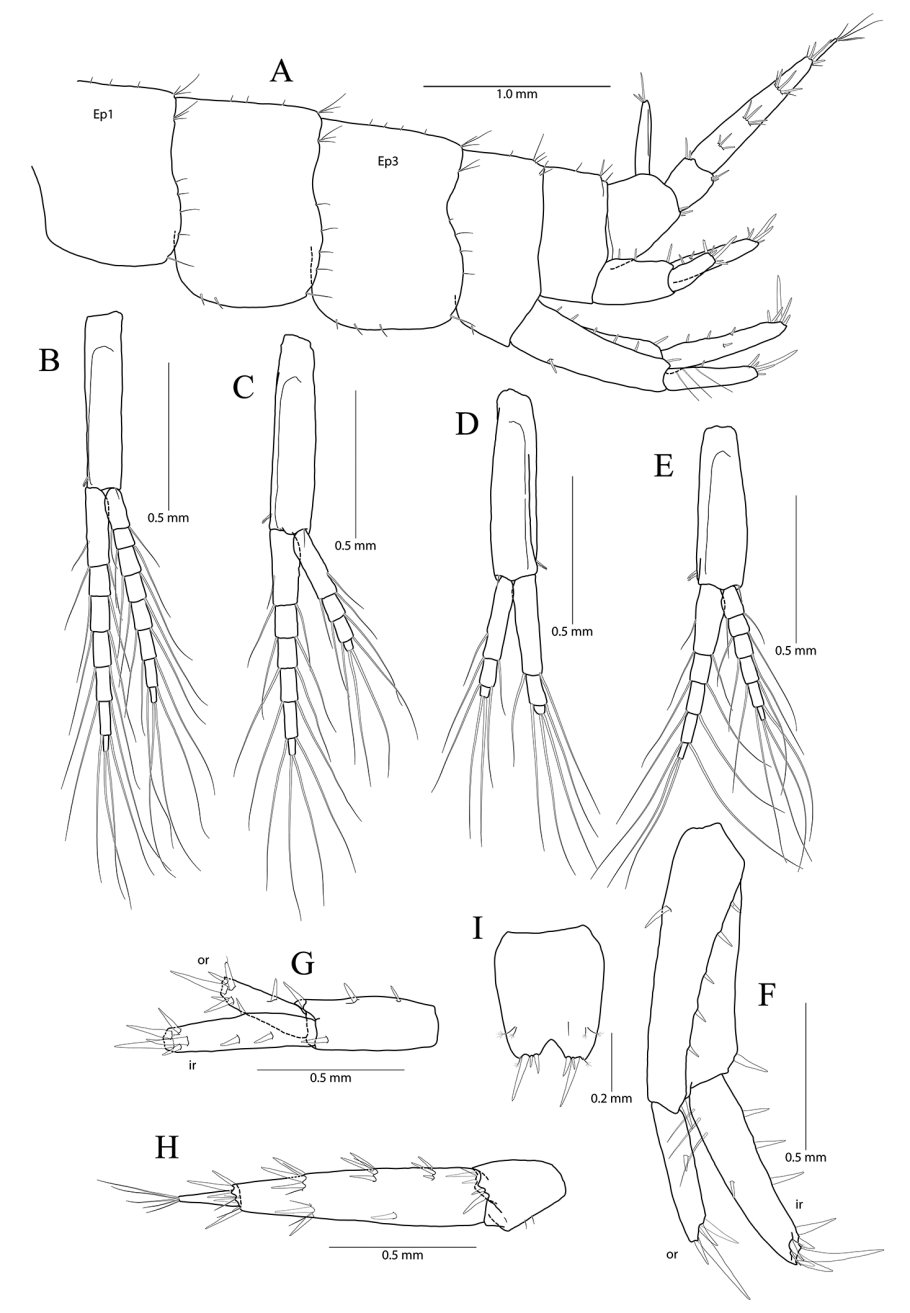
*Pseudocrangonyx minutus* sp. nov. Holotype: female, NIBRV0000862810, 9.1 mm, from Gageo Is., South Korea. **A** epimeral plates and urosomites **B** pleopod 1, left **C** pleopod 2, left **D** pleopod 2, right **E** pleopod 3, left **F** uropod 1 **G** uropod 2 **H** uropod 3 **I** telson. Abbreviations: Ep, epimeral plate; ir, inner ramus; and or, outer ramus.

Figs 13 , 14 , 15 , 16

###### Korean name.

Jak-eun-dong-gul-yeop-sae-u, new

###### Type locality.

Gageodo-ri, Heuksan-myeon, Sinan-gun, Jeollanam-do, South Korea; 34°03'52.2"N, 125°06'55.7"E; an old tube well for using groundwater.

**Material examined**. Type material. ***Holotype***: 1 adult female, 9.1 mm, NIBRIV0000862810. ***Paratypes***: 2 males and 2 females, NIBRIV0000872412. All type materials collected on 7 Oct 2009 by Dr. M-S Kim.

###### Etymology.

The specific name originates from the Latin word *minutus* meaning small, petty. This name refers to more reduced pleopod articles compare to other pseudocrangonyctids.

###### Diagnosis.

Antenna 1, 0.33 × as long as body; flagellum composed of 15 articles. Antenna 2 flagellum composed of seven articles. Both mandibles bearing five raker setae. Maxilla 1 inner lobe with four plumose setae on apical margin; 2^nd^ palp article with six robust setae along distomedial to apical margins, with one oblique row of three setae subdistally. Maxilla 2 inner lobe with one oblique row of ten plumose setae on surface. Gnathopods 1 and 2 each possessing carpus with one and two rastellate setae, respectively. Pereopod 6, 1.33 × as long as pereopod 5; coxa anterior lobe with one seta on ventral margin. Sternal gills absent. Epimeral plate 2 ventral margin with two submarginal setae anteriorly, posterodistal corner slightly notched bearing one elongate seta. Epimeral plate 3 ventral margin with three submarginal setae anteriorly, slightly concaved at the middle, posterodistal corner slightly notched bearing one elongate seta. Pleopods rami reduced. Telson cleft for 0.14% of length.

###### Description.

Holotype female: Body approximately 9.1 mm long. Sternal gills absent (Fig. 13A).

Head (Fig. 13B) 1.26 × as long as pereonite 1; rostrum reduced, with minute setae apically; lateral cephalic lobe anteriorly expanded, apex rounded, slightly dilated anteroventrally; antennal sinus not deep; eye absent.

Antenna 1 (Fig. 13C) 0.33 × as long as body; 1^st^–3^rd^ peduncular articles length ratio of 1.00 : 0.66 : 0.37; 1^st^ article stout, posterior margin with one pair of robust setae and one single robust seta; accessory flagellum bi-articulate, last article very reduced; flagellum 1.14 × as long as peduncles, composed of 15 articles, calceoli absent, aesthetascs present from 3^rd^ to 14^th^ articles.

Antenna 2 (Fig. 13D) 0.59 × as long and more setose than antenna 1; antennal cone well developed, apex rounded; 4^th^ peduncular article margins subparallel, 0.34 × wider than long, posterior margin slightly widening distally and notched at distal corner, 5^th^ article 1.07 × as long as 4^th^ article, three calceoli present on medial surface; flagellum composed of seven articles, 1.13 × as long as 5^th^ peduncular article, single calceoli present from 1^st^ to 3^rd^ articles, aesthetascs absent.

Upper lip (Fig. 13E) rounded, apex slightly produced apically, covered with minute setae, not notched.

Lower lip (Fig. 13F) inner lobes indistinct; outer lobes covered with minute setae; mandibular processes developed.

Mandible (Fig. 13G, H) incisor 5-dentate on both sides; lacinia mobilis tri-cuspidate (two of them finely dentate and one small, produced upwards) on right and 5-dentate on left side; five raker setae present on both sides; molar process columnar, triturative, with one plumose seta on right side only; palp tri-articulate; 2^nd^ article convex and with ten setae medially; 3^rd^ article subfalcate, as long as 2^nd^ article, lined with 18 setae from medial margin to apex.

Maxilla 1 (Fig. 13I) inner lobe subrhomboid, with four plumose setae on apical margin; outer lobe with seven dentate robust setae; palp bi-articulate, 2^nd^ article apex exceeding apical setae of outer plate, with six robust setae along distomedial to apical margins, with one oblique row of three setae subdistally.

Maxilla 2 (Fig. 13J) inner lobe slightly shorter but wider than outer lobe, with one oblique row of ten plumose setae on surface and two rows of simple setae on apical margin; outer lobe apical margin with two rows of simple setae.

Maxilliped (Fig. 13K) inner lobe subrectangular, apex rounded, with six subdentate robust setae mediodistally and with seven plumose setae subapically; outer lobe elongate semicircular, 1.13 × as long as 2^nd^ palp article, with five robust setae and nine plumose setae apical or subapically; inner lobe apex not exceeding middle of 2^nd^ article of palp, with three plumose setae and five dentate robust setae apically; palp composed of four articles; 2^nd^ article with many setae on medial margin; 3^rd^ article slightly dilated distally, 0.5 × as long as 2^nd^ article; 4^th^ article falcate, 0.76 × as long as 3^rd^ article, apical setae 0.75 × as long as 4^th^ article.

Gnathopod 1 (Fig. 14A, B) coxa subrectangular, 1.43 × wider than long, anterodistal corner somewhat produced, with five setae marginally, ventral margin a little convex; basis obtuse trapezoidal, posteriorly expanded, 0.51 × wider than long, lined with elongate simple setae posteriorly, anterior margin without setae; ischium 0.16 × as long as basis, with small anterior lobe; carpus 0.58 × as long as basis, with two robust seta on anterior margin, carpal lobe not developed, apex rounded with one rastellate seta and many simple or serrate setae; propodus subovate, 1.32 × as long and 1.48 × as wide as basis, posterior margin distal half with four robust setae successively increasing distally, palm irregular, defined by largest lateral seta of posterior margin, finely serrated, lined with six robust setae medially; dactylus as long as palm, inner margin toothed, outer margin with two setae, unguis developed.

Gnathopod 2 (Fig. 14C–E) as long as gnathopod 1; coxa 1.32 × as long as that of gnathopod 1, 1.22 × wider than long, , anterior and ventral margins not produced, rounded, lined with 15 setae, posterior margin slightly humped proximally; basis posteroproximally expanded, 0.34 × wider than long, lined with elongate simple setae posteriorly, anterior margin without setae; ischium 0.14 × as long as basis, with small anterior lobe; carpus 0.56 × as long as basis, with two pairs of robust and simple setae on anterior margin, carpal lobe not developed, broader than that of gnathopod 1, margin weakly crenulate, with two rastellate setae and many simple or serrate setae; propodus trapezoidal, 1.00 × as long and 1.54 × as wide as basis, anterior margin slightly convex, posterior margin 0.57 × as long as anterior margin, lined with five clusters of elongate setae, with two defining robust setae distally; palm irregular, finely serrated, lined with nine medial and eleven lateral robust setae (two defining setae stouter than others); dactylus as long as palm, inner margin toothed, outer margin with one seta, unguis developed.

Pereopod 3 (Fig. 15A, B) coxa subrectangular, 1.53 × wider than long, slightly expanded posteroventrally, with six setae anteriorly, ventral margin concave, with three posterior setae; basis expanded, 0.63 × as wider than long, width longest at proximal 1/3, anterior margin lined with minute setae, with one sensory seta, posterior margin with seven elongate setae; merus anterodistally expanded, 0.32 × wider than long, 0.62 × as long as basis, anterodistal corner produced, apex blunt; carpus not expanded, 0.50 × as long as basis; propodus linear, as long as carpus; dactylus 0.40 × as long as propodus, unguis developed.

Pereopod 4 (Fig. 15C, D) similar to pereopod 3 except that merus 0.88 × as long as that of pereopod 3; different number or position of several setae.

Pereopod 5 (Fig. 15E) coxa bilobate, anterior lobe larger than posterior lobe, expanded ventrally (0.81 × longer than wide), margin rounded, lined with five simple setae ventrally; posterior lobe with one seta at posterior corner; basis expanded, subrectangular, 0.53 × wider than long, anterior margin slightly convex, lined with five single robust setae, posterior margin more expanded, lined with eleven simple setae, distal corner produced forming an angle; merus posterodistally expanded, 0.42 × as wide and 0.73 × as long as basis, with three pairs of simple setae on anterior margin and two robust setae on posterior margin; carpus subrectangular, posterodistal corner slightly produced, 0.25 × wider than long, 0.61 × as long as basis, with two anterior and one posterior clusters of simple and robust setae, with well-developed distal clusters of setae at both anterior and posterior corners (longest seta 0.31× as long as propodus); propodus linear, 1.17 × as long as carpus, anterior margin with two clusters of simple and robust setae (longest seta of distal cluster slightly not exceeding end of propodus) and one pair of locking robust setae distally, posterior margin with one marginal short seta and one distal cluster of elongate setae; dactylus 0.31 × as long as propodus, unguis developed.

Pereopod 6 (Fig. 15F, G) 1.33 × as long as pereopod 5; coxa bilobate, anterior lobe 0.71 × as long as that of pereopod 5, ventral margin with one seta only, posterior lobe expanded backward, with one seta at posterior corner; basis expanded, subrectangular, 1.12 × as long and 1.00 × as wide as that of pereopod 5, 0.49 × wider than long, posterodistal corner produced forming an angle; merus posterodistally expanded, 0.44 × as wide and 0.85 × as long as basis, with three anterior and two posterior setal clusters; carpus rectangular, 0.23 × wider than long, 0.80 × as long as basis, with two setal clusters on anterior and posterior margins, respectively, with well-developed antero- and posterodistal setal clusters (longest seta 0.25 × as long as propodus); propodus linear, 1.09 × as long as carpus, anterior margin with three setal clusters (longest seta of distal cluster not exceeding end of propodus) and one pair of locking robust setae distally, posterior margin with one marginal short seta and one distal cluster of elongate setae; dactylus 0.33 × as long as propodus, unguis developed.

Pereopod 7 (Fig. 15H, I) 1.01 × as long as pereopod 6; coxa unilobed, subtriangular, 0.83 × as long as that of pereopod 6, posteriorly expanded with one seta at posterior corner, with one seta on ventral margin; basis expanded, subrectangular, 1.09 × as long and 0.93 × as wide as that of pereopod 6, 0.42 × wider than long, posterodistal corner slightly produced but weaker than those of pereopods 6 and 7; merus posterodistally expanded, 0.60 × as wide and 0.70 × as long as basis, with two setal clusters on anterior and posterior margins, respectively; carpus rectangular, 0.26 × wider than long, 0.70 × as long as basis, with one cluster and three clusters of setae on anterior and posterior margins, respectively, with well-developed antero- and posterodistal setal clusters (longest seta 0.25 × as long as propodus); propodus linear, 1.09 × as long as carpus, anterior margin with three setal clusters (longest seta of distal cluster shorter than that of pereopod 6) and one pair of locking robust setae distally, posterior margin with one marginal short seta and one distal cluster of elongate setae (those shorter than that of pereopod 6); dactylus 0.42 × as long as propodus, unguis developed.

Epimeral plate 1 subquadrate, ventral margin without setae, posterior margin convex, lined with four setae, posterodistal corner slightly notched bearing one elongate seta. Epimeral plate 2 subquadrate, larger than plate 1, ventral margin with two submarginal setae anteriorly, posterior margin convex, with four setae, posterodistal corner slightly notched bearing one elongate seta. Epimeral plate 3 larger than plate 2, ventral margin with three submarginal setae anteriorly, slightly concaved at the middle, posterodistal corner slightly notched bearing one elongate seta (Fig. 16A).

Pleopod 1 (Fig. 16B) peduncle with one pair of retinaculae mediodistally, one simple seta laterodistally; outer ramus 1.23 × as long as peduncle, composed of eight articles; inner ramus 1.49 × as long as peduncle, composed of seven articles (coalesced 1^st^ article exceeding proximal two articles of outer ramus combined).

Pleopod 2 (Fig. 16C, D) peduncle 1.10 × as long as that of pleopod 1, with one pair of retinaculae mediodistally and one simple seta laterodistally; reduction of rami different from right and right sides; in left side, outer ramus 0.66 × as long as peduncle, composed of four articles; inner ramus 1.16 × as long as peduncle, composed of six articles (coalesced 1^st^ articles on inner and outer rami equal each other); in right side, both rami more reduced or coalesced.

Pleopod 3 (Fig. 16E) 0.80 × as long as pleopod 2; peduncle 0.84 × as long as that of pleopod 2, with one pair of retinaculae mediodistally and one seta laterodistally; outer ramus 0.84 × as long as peduncle, composed of six articles; inner ramus 1.06 × as long as peduncle, composed of five articles (coalesced 1^st^ article 1.40 × as long as proximal two articles of outer ramus combined).

Uropod 1 (Fig. 16F) peduncle with one basofacial seta, with five margin robust setae and one distal seta dorsolaterally, with one distal robust seta dorsomedially; outer ramus 0.51 × as long as peduncle, with one distal robust seta dorsomedially, apical cluster composed of five robust setae (longest seta 0.59 × as long as outer ramus); inner ramus 0.70 × as long as peduncle, with three robust setae dorsomedially and one robust seta dorsolaterally, with three elongate setae on ventral margin subproximally, apical cluster of setae composed of seven robust setae and one sensory seta (longest seta 0.45 × as long as inner ramus).

Uropod 2 (Fig. 16G) 0.57 × as long as uropod 1; peduncle 0.46 × as long as that of uropod 1, with two marginal robust setae and one distal robust seta dorsolaterally, with one distal robust seta dorsomedially; outer ramus 0.73 × as long as peduncle, with one distal robust seta dorsolaterally, apical cluster composed of five robust setae (longest seta 0.61 × as long as outer ramus, one of those with abnormal apex); inner ramus 1.15 × as long as peduncle, with two robust setae dorsomedially and one robust seta dorsolaterally, apical cluster composed of five robust setae and one sensory seta (longest seta 0.38× as long as inner ramus).

Uropod 3 (Fig. 16H) uniramous, 0.76 × as long as uropod 1; peduncle short, 0.62 × as long as uropod 2, with two minute setae on medial margin, with two setal clusters laterodistally; ramus 3.75 × as long as peduncle, bi-articulate, proximal article gradually diminished in width, with four clusters of setae laterally, with two clusters of setae and one single robust seta medially, (longest seta of distal cluster not exceeding distal article ramus), distal article 0.24 × as long as proximal article, with five elongate simple setae apically.

Telson (Fig. 16I) 0.81 × as wide as long, cleft for 14% of length, each lobe with one pair of penicillate setae dorsally, and one penicillate seta and three robust setae on apex.

###### Remarks.

*Pseudocrangonyx minutus* sp. nov. is very similar to *P. daejeonensis* Lee, Tomikawa, Nakano & Min, 2018 in that the telson is concave (less than 15%) at the apex. However, the lateral cephalic lobe is more produced anteriorly and the antennal sinus is deeper in *P. minutus* compared to *P. daejeonensis* (Lee et al. 2018). In addition, the 2^nd^ peduncular article of antenna 1 is 0.66 times as long as 1^st^ article (compared to 0.5 times in *P. daejeonensis*), the flagellum of antenna 1 is composed of 15 articles (compared to ten articles in *P. daejeonensis*), antenna 2 has calceoli on the medial surface of the 5^th^ peduncular article and the 1^st^–3^rd^ flagellum articles (which are absent in *P. daejeonensis*), the flagellum of antenna 1 is composed of seven articles (compared to four articles in *P. daejeonensis*), eight raker setae are present on each mandible (compared to three on left and two on right in *P. daejeonensis*), the mandibular palp is more setose, the inner lobe of maxilla 1 has four plumose setae on the apical margin (compared to two plumose setae in *P. daejeonensis*), the 2^nd^ article of palp maxilla 1 shows six dentate robust setae apically (two dentate robust setae in *P. daejeonensis*), and the outer plate of the maxilliped is larger (Lee et al. 2018). Moreover, in *P. minutus*, each basis of gnathopods 1 and 2 is more setose posteriorly and each propodus is less expanded posteriorly, pereopods 3 and 4 are also more setose, each propodus of pereopods 5–7 is slender and has more robust setae than that of *P. daejeonensis*, each posterior margin of the bases in pereopods 5–7 has a smooth or smaller angle at the distal corner, each propodus of pereopods 5–7 is more slender and elongate. Lastly, in *P. minutus* sp. nov., the peduncle of uropod 1 has five robust setae marginally on the dorsolateral margin (compared to two robust setae in *P. daejeonensis*) and the inner ramus of uropod 1 has three elongate simple setae proximally on the ventral margin (compared to one seta in *P. daejeonensis*) (Lee et al. 2018).

*Pseudocrangonyx minutus* sp. nov. is also similar to *P. komaii* Tomikawa & Nakano, 2018 from Japan with regard to general shape and length ratio of the articles in the gnathopods and pereopods, the reduced number of the articles of rami in the pleopods, and the slightly concave shape of the telson (less than 15%) at the apex. On the other hand, *P. minutus* sp. nov. can be readily distinguished from *P. komaii* by the slender and elongate peduncular articles of antennae 1 and 2, the six apical robust setae in the palp of maxilla 1 (compared to four in *P. komaii*), by one and two rastellate setae in gnathopods 1 and 2, respectively (which are absent in *P. komaii*), by a more setose posterior margin of the basis in each gnathopod, by the presence of five setae ventrally on the anterior lobe of coxa 5 (compared to one in *P. komaii*), and by the more expanded basis of pereopod 5 (Tomikawa and Nakano 2018).

##### Pseudocrangonyx villosus sp. nov.

Taxon classificationAnimaliaAmphipodaPseudocrangonyctidae

0FDA7021-B8DA-5D35-A2FE-27FB2AC3C01E

http://zoobank.org/05766F5F-4020-49AE-975D-C2AE60663AD4

**Figure 17.**
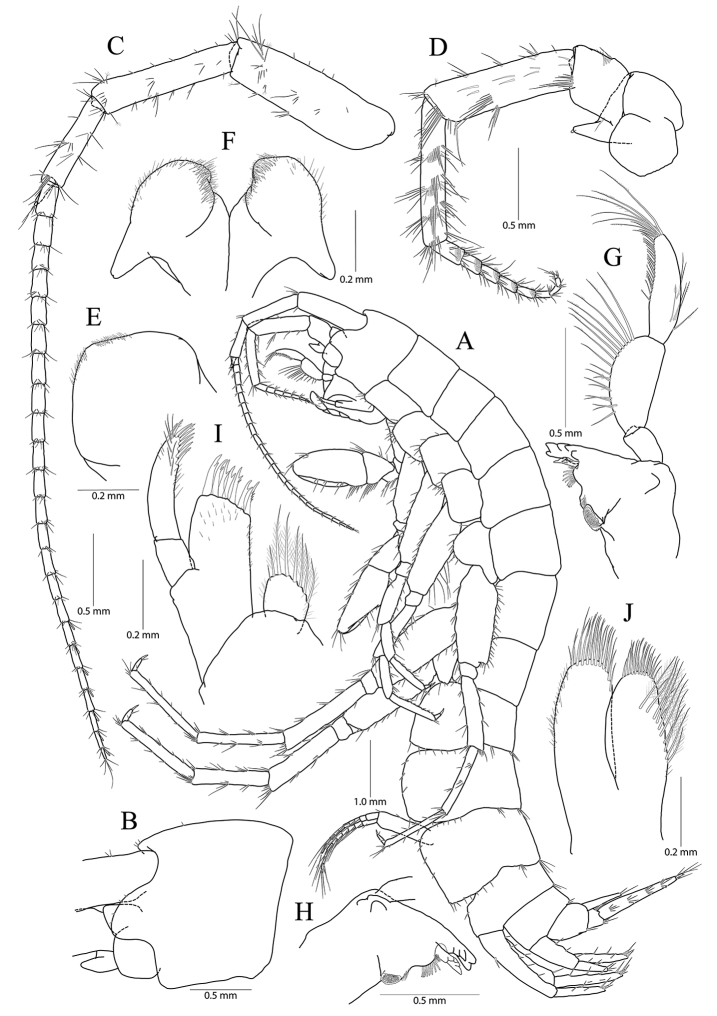
*Pseudocrangonyx villosus* sp. nov. Holotype: female, NIBRV0000862811, 12.4 mm, from Ansanan Cave, South Korea. **A** habitus **B** head **C** antenna 1 **D** antenna 2 **E** upper lip **F** lower lip **G** right mandible **H** left mandible **I** maxilla 1 **J** maxilla 2.

**Figure 18.**
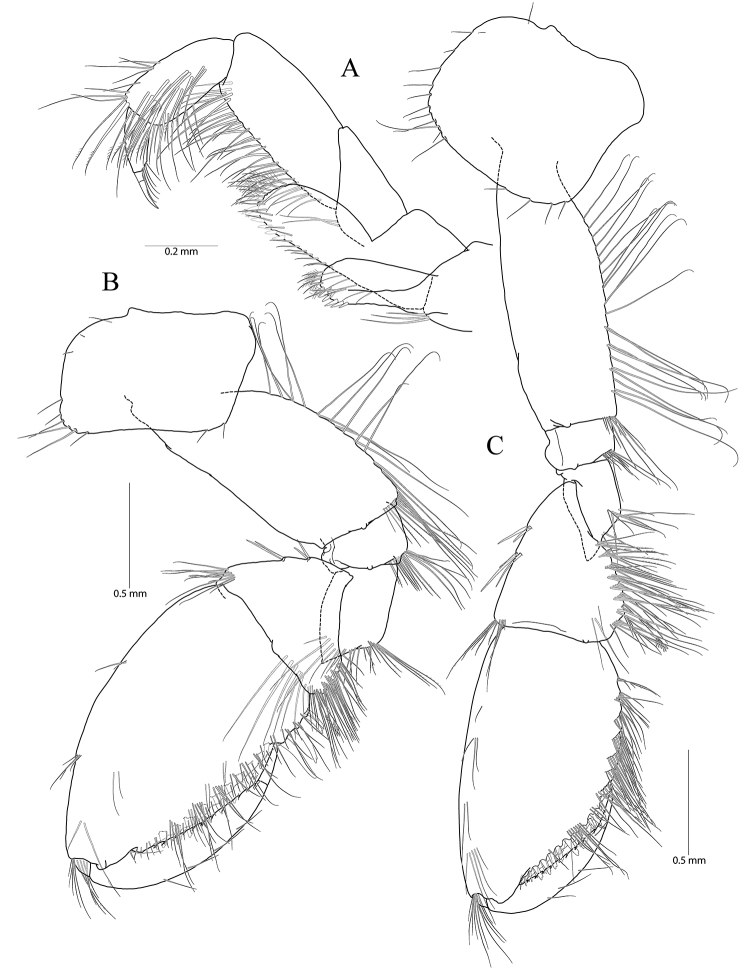
*Pseudocrangonyx villosus* sp. nov. Holotype: female, NIBRV0000862811, 12.4 mm, from Ansanan Cave, South Korea. **A** maxilliped **B** gnathopod 1 **C** gnathopod 2.

**Figure 19.**
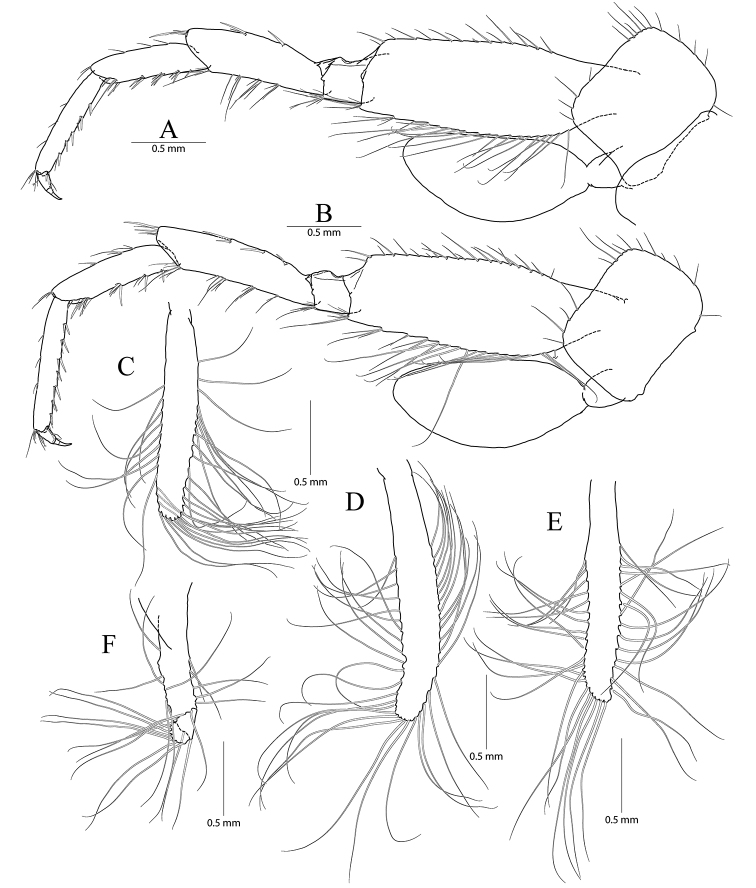
*Pseudocrangonyx villosus* sp. nov. Holotype: female, NIBRV0000862811, 12.4 mm, from Ansanan Cave, South Korea. **A** pereopod 3 **B** pereopod 4 **C–F** oostegites of gnathopod 2–pereopod 5.

**Figure 20.**
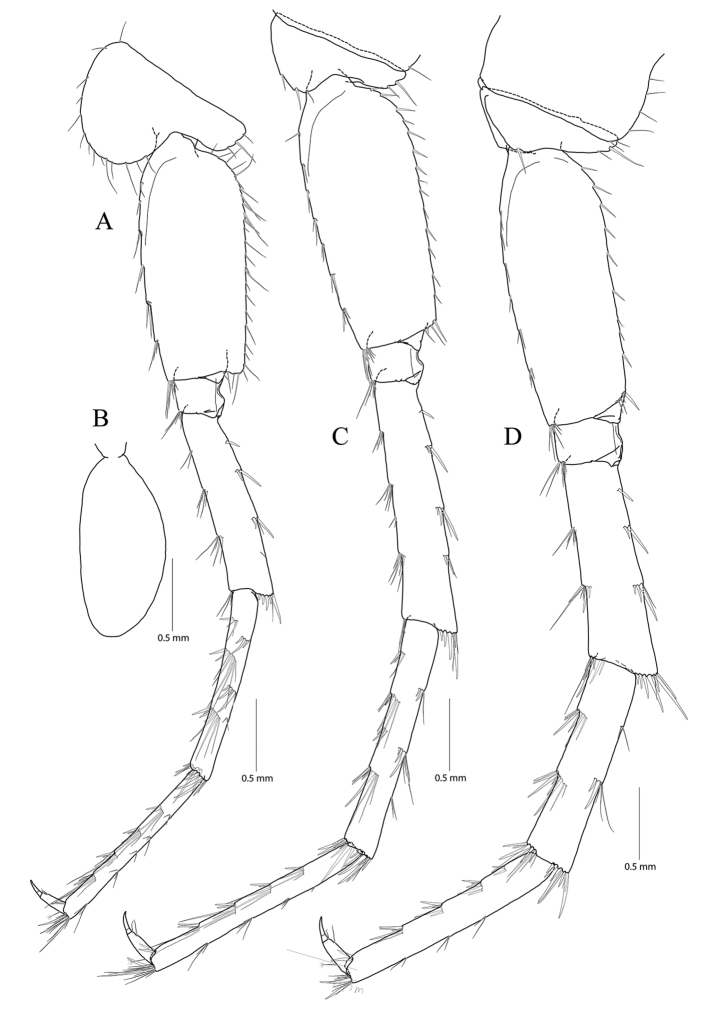
*Pseudocrangonyx villosus* sp. nov. Holotype: female, NIBRV0000862811, 12.4 mm, from Ansanan Cave, South Korea. **A** pereopod 5 **B** coxal gill of pereopod 5 **C** pereopod 6 **D** pereopod 7.

**Figure 21.**
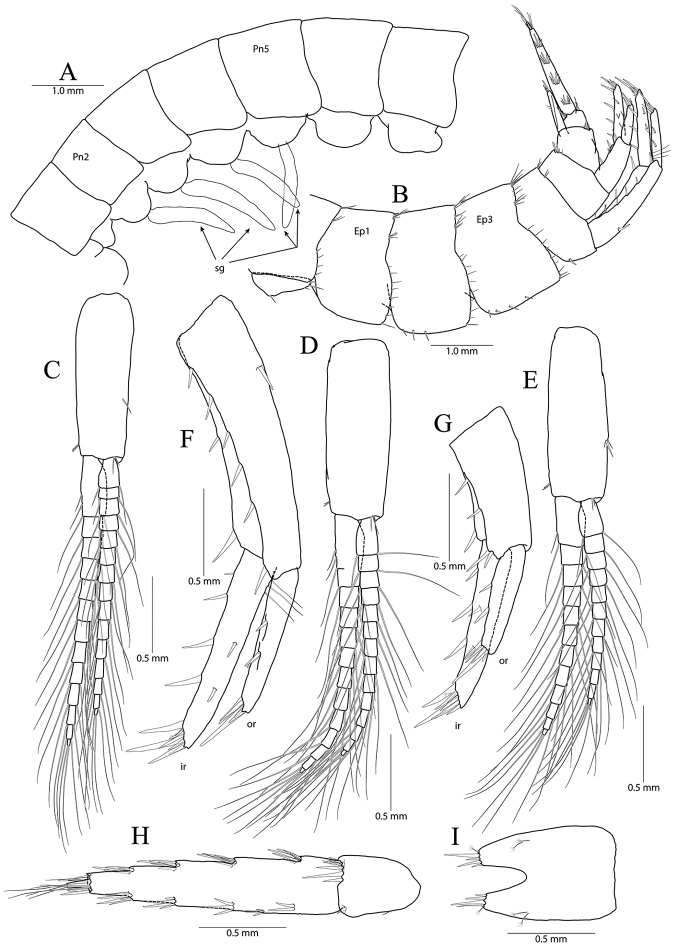
*Pseudocrangonyx villosus* sp. nov. Holotype: female, NIBRV0000862811, 12.4 mm, from Ansanan Cave, South Korea. **A** sternal gills **B** epimeral plates and urosomites **C** pleopod 1 **D** pleopod 2 **E** pleopod 3 **F** uropod 1 **G** uropod 2 **H** uropod 3 **I** telson. Abbreviations: Ep, epimeral plate; Pn, pereonite; sg, sternal gill; ir, inner ramus; and or, outer ramus.

Figs 17 , 18 , 19 , 20 , 21

###### Korean name.

Teol-son-dong-gul-yeop-sae-u, new

###### Type locality.

Ansanangul Cave, Beolcheon-ri, Danseong-myeon, Danyang-gun, Chungcheongbuk-do, South Korea; 36°52'10"N, 128°17'00"E.

**Material examined.** Type material. ***Holotype***: 1 female, 12.4 mm, NIBRIV0000862811. ***Paratypes***: 2 specimens, NIBRIV0000872413. All type materials were collected on 17 Mar 2002 by YG Choi.

###### Etymology.

The specific name originates from the Latin word *villosus* meaning hairy. This name refers to the more setose bases of pereopods 3 and 4 compared to other pseudocrangonyctids.

###### Diagnosis.

Antenna 1, 1.61 × as long as body; 1^st^–3^rd^ peduncular articles length ratio of 1.00 : 0.86 : 0.64. Antenna 2 peduncles moderate. Mandibles eight raker setae present on both sides. Maxilla 1 inner lobe with seven plumose setae on apical margin; palp with nine robust setae along distomedial to apical margins, with one oblique row of seven setae subdistally. Maxilla 2 with one oblique row of ten plumose setae on surface. Gnathopods 1 and 2 each possessing carpus with two rastellate setae, respectively. Pereopods 3 and 4 basis more setose on both margins. Pereopods 5–7 basis slightly elongate. Pereopod 5 carpus and propodus with rows of elongate setae medially. Pereopod 6, 1.24 × as long as pereopod 5. Pereopod 7 merus, carpus and propodus stout. Sternal gills present from pereonites 2 to 5 (1+1+1+1 in formulae). Epimeral plates 2 ventral margin with three setae anteriorly, posterodistal corner slightly notched bearing one elongate seta. Epimeral plates 3 posterior margin with eight setae, posterodistal corner slightly notched bearing one elongate seta, ventral margin with four submarginal setae anteriorly. Uropod 1 inner ramus with three elongate simple setae on ventral margin subproximally. Telson 0.71 × as wide as long, cleft for 35% of length.

###### Description.

Holotype female: Body (Fig. 17A) approximately 12.4 mm long.

Head (Fig. 17B) as long as pereonite 1; rostrum reduced, with three minute setae; lateral cephalic lobe anteriorly expanded, apex rounded, slightly dilated anteroventrally; antennal sinus not deep; eye absent.

Antenna 1 (Fig. 17C) 0.61 × as long as body; 1^st^–3^rd^ peduncular articles length ratio of 1.00 : 0.86 : 0.64; 1^st^ article stout, posterior margin with simple setae only; accessory flagellum bi-articulate, last article very reduced; flagellum 1.32 × as long as peduncles, composed of 23 articles, calceoli absent, aesthetascs present from 16^th^ to last articles.

Antenna 2 (Fig. 17D) 0.59 × as long as antenna 1; antennal cone developed, apex rounded; 4^th^ peduncular article margins parallel, 0.25 × wider than long, posterior margin with one row of setae in the middle and one elongate seta subdistally, 5^th^ article 0.95 × as long and 0.61 × as wide as 4^th^ article, three calceoli present on medial surface; flagellum composed of nine articles, 1.05 × as long as 5^th^ peduncular article, single calceoli present from 1^st^ to 5^th^ articles medially, aesthetascs absent.

Upper lip (Fig. 17E) rounded, apex slightly produced apically, covered with minute setae, not notched.

Lower lip (Fig. 17F) inner lobes indistinct; outer lobes covered with minute setae; mandibular processes developed.

Mandible (Fig. 17G, H) incisor 5-dentate on both sides; lacinia mobilis tri-cuspidate (two of them finely dentate and one small, produced upwards) on right and 5-dentate on left side; eight raker setae present on both sides; molar process columnar, triturative, without plumose setae on both sides; palp tri-articulate; 2^nd^ article convex and with 18 setae medially; 3^rd^ article subfalcate, as long as 2^nd^ article, lined with 27 setae from medial margin to apex, with six setae laterally.

Maxilla 1 (Fig. 17I) inner lobe subrhomboid, with seven plumose setae on apical margin; outer lobe with seven dentate robust setae; palp bi-articulate, 2^nd^ article apex exceeding apical setae of outer plate, with nine robust setae along distomedial to apical margins, with one oblique row of seven setae subdistally.

Maxilla 2 (Fig. 17J) inner lobe slightly shorter but wider than outer lobe, with one oblique row of ten plumose setae on surface and two rows of simple setae on apical margin; outer lobe apical margin with two rows of simple setae.

Maxilliped (Fig. 18A) inner lobe subrectangular, apex rounded, with six subdentate robust setae mediodistally and with seven plumose setae apically and subapically; outer lobe elongate semicircular, as long as 2^nd^ palp article, with six plumose setae on apical margin and six dentate robust setae on mediodistal margin; palp composed of four articles; 2^nd^ article with many setae on medial margin; 3^rd^ article slightly dilated distally, 0.63 × as long as 2^nd^ article; 4^th^ article falcate, 0.59 × as long as 3^rd^ article, apical setae 0.71 × as long as 4^th^ article.

Gnathopod 1 (Fig. 18B) coxa subrectangular, 1.43 × wider than long, anterodistal corner somewhat produced, with six setae marginally, ventral margin a little convex, posterior margin slightly humped proximally; basis obtuse trapezoidal, posteriorly expanded, 0.51 × wider than long, lined with elongate simple setae posteriorly, anterior margin without setae; ischium 0.26 × as long as basis, with small anterior lobe; carpus 0.56 × as long as basis, with two pairs of robust setae on anterior margin, carpal lobe not developed, apex rounded with two rastellate setae and many simple or serrate setae; propodus subovate, 1.32 × as long and 1.48 × as wide as basis, posterior margin with four robust setae successively increasing distally, palm irregular, defined by largest lateral seta of posterior margin, finely serrated, lined with ten robust setae medially; dactylus as long as palm, inner margin toothed, outer margin with four setae, unguis developed.

Gnathopod 2 (Fig. 18C) 1.10 × as long as gnathopod 1; coxa 1.13 × as long as that of gnathopod 1, 1.22 × wider than long, anterior and ventral margins not produced, rounded, lined with 15 setae, posterior margin humped proximally; basis somewhat expanded posteroproximally, 0.34 × wider than long, lined with elongate simple setae posteriorly, anterior margin without setae; ischium 0.14 × as long as basis, with small anterior lobe; carpus 0.56 × as long as basis, with two clusters of robust setae on anterior margin, carpal lobe not developed, broader than that of gnathopod 1 margin, weakly crenulate, with two rastellate setae and many simple or serrate setae; propodus trapezoidal, 1.00 × as long and 1.54 × as wide as basis, anterior margin slightly convex, posterior margin 0.57 × as long as anterior margin, lined with seven clusters of elongate setae, with two defining robust setae distally; palm irregular, finely serrated, lined with 12 medial and 14 lateral robust setae; dactylus as long as palm, inner margin toothed, outer margin with three setae, unguis developed.

Pereopod 3 (Fig. 19A) coxa subrectangular, 1.19 × wider than long, slightly humped posteroproximally, ventral margin a little concave, with eleven setae anteriorly and four setae at posteroventral corner; coxal gill subovate; basis expanded, 0.69 × as wide and 3.05 × as long as coxa, width longest at proximal 1/3 and gradually diminished distally, margins lined with setae but posterior ones longer than anterior ones; ischium 0.16 × as long as basis; merus anterodistally expanded, 0.30 × wider than long, 0.49 × as long as basis, anterodistal corner produced, apex blunt; carpus not expanded, 0.37 × as long as basis; propodus, 0.42 × as long as basis, linear; dactylus 0.32 × as long as propodus, unguis developed.

Pereopod 4 (Fig. 19B) similar to pereopod 3; merus, carpus and propodus slightly longer than those of pereopod 3.

Pereopod 5 (Fig. 20A, B) coxa bilobate, anterior lobe larger than posterior lobe, expanded ventrally (1.00 × as long as wide), margin rounded, lined with 13 simple setae; posterior lobe with three setae posteriorly; coxal gill subovate; basis expanded, subrectangular, slightly expanded, 0.43 × wider than long, anterior margin slightly convex, lined with two single robust setae and four clusters of robust and simple setae, posterior margin lined with 28 simple setae, distal corner produced forming an angle; merus posterodistally expanded, 0.37 × as wide and 0.76 × as long as basis, with three clusters of simple setae on anterior margin and one robust seta and two clusters of robust setae on posterior margin; carpus not expanded, 0.79 × as long as basis, with three setal clusters on medial surface anteriorly and two robust setal clusters on lateral surface posteriorly; propodus linear, as long as carpus, anterior margin with four setal clusters (longest seta of distal cluster slightly exceeding end of propodus) and one pair of locking robust setae distally, posterior margin with five clusters of simple setae, with one cluster of simple and robust setae at distal corner; dactylus 0.26 × as long as propodus, unguis developed.

Pereopod 6 (Fig. 20C) 1.24 × as long as pereopod 5; coxa bilobate, anterior lobe 0.59 × as long as that of pereopod 5, with three setae ventrally, posterior lobe narrow, expanded backward, with two setae posteriorly; basis expanded, subrectangular, slightly expanded, 1.16 × as long and 1.07 × as wide as that of pereopod 5, 0.40 × wider than long, with three single and four clusters of setae on anterior margin, with 16 setae on posterior margin, posterodistal corner produced forming an angle, but weaker than that of pereopod 5; merus posterodistally expanded, 0.48 × as wide and 0.89 × as long as basis, with four anterior and four posterior setal clusters; carpus not expanded, 0.83 × as long as basis, with four setal clusters on medial surface anteriorly and three setal clusters on lateral surface posteriorly; propodus linear, as long as carpus, anterior margin with four setal clusters (longest seta of distal cluster not exceeding end of propodus) and one pair of locking robust setae distally, posterior margin with one single seta and three clusters of simple setae and one distal cluster of elongate setae; dactylus 0.27 × as long as propodus, unguis developed.

Pereopod 7 (Fig. 20D) 0.93 × as long as pereopod 6, but width slightly thicker universally; coxa unilobed, subtriangular, 0.82 × as long as that of pereopod 6, posteriorly expanded with three setae at posterior corner, with one seta on ventral margin; basis expanded, subrectangular, as long and wide as that of pereopod 6, posterodistal expansion weaker than those of pereopods 6 and 7; merus posterodistally expanded, 0.67 × as wide and 0.77 × as long as basis, with two setal clusters anteriorly and one single robust seta and two setal clusters posteriorly; carpus as long as merus, rectangular, not narrowed, 0.24 × as wide as long; propodus linear but thicker than that of pereopod 6, 1.10 × as long as carpus, anterior margin with four setal clusters (without elongate setae) and one pair of locking robust setae distally, with three clusters of simple setae posteriorly; dactylus 0.30 × as long as propodus, unguis developed.

Oostegites (Fig. 19C–F) present from gnathopod 2 to pereopod 5, narrow, with marginal seta, shortest in that of pereonite 5.

Sternal gills (Fig. 21A) present from pereonites 2 to 5 (1+1+1+1 in formulae), narrower than oostegites, similar to each other in length.

Epimeral plate 1 subquadrate, a little produced posteroventrally, ventral margin without setae, posterior margin with seven setae, posterodistal corner slightly notched bearing one elongate seta. Epimeral plate 2 subquadrate, slightly larger than epimeron 1, ventral margin with three setae anteriorly, posterior margin with nine setae, posterodistal corner slightly notched bearing one elongate seta. Epimeral plate 3 posterior margin with eight setae, posterodistal corner slightly notched bearing one elongate seta, ventral margin with four submarginal setae anteriorly (Fig. 21B).

Pleopod 1 (Fig. 21C) peduncle with one pair of retinaculae mediodistally and one pair of simple setae laterodistally, with one seta on lateral margin; outer ramus 1.55 × as long as peduncle, composed of 15 articles; inner ramus 1.74 × as long as peduncle, composed of 13 articles (coalesced 1^st^ article as long as proximal three articles of outer ramus combined).

Pleopod 2 (Fig. 21D) peduncle 1.10 × as long as that of pleopod 1, with one pair of retinaculae mediodistally and one pair of simple setae laterodistally, margins without setae; outer ramus 1.33 × as long as peduncle, composed of 14 articles; inner ramus 1.10 × as long as outer ramus, composed of twelve articles (coalesced 1^st^ article with suture, as long as proximal three articles of outer ramus combined).

Pleopod 3 (Fig. 21E) 0.92 × as long as pleopod 2; peduncle 0.98 × as long as that of pleopod 2, with one pair of retinaculae mediodistally and one pair of simple setae laterodistally, with one pair of setae on lateral margin; outer ramus 1.18 × as long as peduncle, composed of twelve articles; inner ramus 1.15 × as long as outer ramus, composed of twelve articles (1^st^ coalesced article reaching middle of 2^nd^ article of outer ramus).

Uropod 1 (Fig. 21F) peduncle with one basofacial seta, with four marginal robust setae and one distal robust seta dorsolaterally, with one marginal robust seta and one distal robust seta dorsomedially; outer ramus 0.53 × as long as peduncle, with one robust seta dorsomedially and two robust setae dorsolaterally, apical cluster composed of five robust setae; inner ramus 1.36 × as long as outer ramus, with four robust setae dorsomedially and two robust setae dorsolaterally, apical cluster composed of seven robust setae and one sensory seta, with three elongate simple setae on ventral margin subproximally.

Uropod 2 (Fig. 21G) 0.68 × as long as uropod 2; peduncle 0.50 × as long as that of uropod 1, with two marginal setae and one pair of distal setae dorsolaterally, with one distal seta dorsomedially; outer ramus 0.78 × as long as peduncle, with two robust setae dorsolaterally, apical cluster composed of five robust setae; inner ramus 1.48 × as long as outer ramus, with three robust setae dorsomedially and two robust setae dorsolaterally, apical cluster composed of seven robust setae and one sensory seta.

Uropod 3 (Fig. 21H) uniramous, 0.86 × as long as uropod 1; peduncle short, 0.57 × as long as uropod 2, with one minute seta on medial margin and one robust seta mediodistally, with one setal cluster on laterodistal margin; ramus 3.24 × as long as peduncle, bi-articulate, proximal article gradually diminished in width, with six clusters of setae laterally and five clusters of setae medially (longest seta of distal cluster exceeding distal article ramus), distal article 0.08 × as long as proximal article, with three elongate simple setae apically.

Telson (Fig. 21I) 0.71 × as wide as long, cleft for 35% of length, each lobe with one pair of penicillate setae dorsally, and one penicillate seta and four robust setae on apex.

###### Remarks.

*Pseudocrangonyx villosus* sp. nov. is distinguished from other Korean pseudocrangonyctids such as *P. asiaticus*, *P. coreanus*, *P. concavus* sp. nov., *P. crassus* sp. nov., *P. daejeonensis*, *P. gracilipes* sp. nov., *P. joolaei*, *P. minutus* sp. nov., and *P. villosus* sp. nov. in that antenna 1 is longer than half length of the body and the 2^nd^ peduncular article is 0.89 times as long as the 1^st^ peduncular article. In addition, maxilla 1 has seven plumose setae on the inner lobe, and a row on the inner lobe of maxilla 2 is composed of ten plumose setae. Both gnathopods have two rastellate setae on the carpus; each basis of pereopods 3 and 4 is more setose along the margins while the bases of pereopods 5–7 are more elongate than those of other Korean pseudocrangonyctids mentioned above. Finally, the sternal gills are present in pereonites 2 to 5 (1+1+1+1 in formulae) and the posterodistal corners of all epimeral plates are notched (Uéno 1934, 1966, Lee et al. 2018, 2020).

### Key to known species of the genus *Pseudocrangonyx* from Korean underground waters [except *P. asiaticus* sensu (Uéno, 1934)]

**Table:** 

1	Telson apex slightly cleft (less than 15%)	**2**
–	Telson apex deeply cleft (more than 15%)	**4**
2	Sternal gill present on pereonites	***P. coreanus* Uéno, 1966**
–	Sternal gill absent on pereonites	**3**
3	Maxilla 1 palp with 6 dentate robust setae apically. Uropod 2 outer ramus with robust setae marginally	***P. minutus* sp. nov.**
–	Maxilla 1 palp with 2 dentate robust setae apically. Uropod 2 outer ramus without robust setae marginally	***P. daejeonensis* Lee, Tomikawa, Nakano & Min, 2018**
4	Pereopods 5 carpus elongate and slender. Epimeral plates 2 and 3 posterodistal corner notched	**5**
–	Pereopod 5 carpus not elongate and slender, slightly expanded. Epimeral plates 2 and 3 posterodistal corner not notched	**6**
5	Maxilla 1 and 2 both inner plates with 4 plumose setae	***P. gracilipes* sp. nov.**
–	Maxilla 1 and 2 each inner plate with 7 and 10 plumose setae, respectively	***P. villosus* sp. nov.**
6	Antenna 2, 4^th^ and 5^th^ peduncular articles not expanded in male. Epimeral plate 3 ventral margin slightly emarginated	***P. concavus* sp. nov.**
–	Antenna 2, 4^th^ and 5^th^ peduncular articles expanded in male. Epimeral plate 3 ventral margin not emarginated	**7**
7	Pereopod 6, anterior lobe of coxa with 2 robust setae ventrally. Uropods 1–2 outer ramus with robust setae on medial margin	***P. joolaei* Lee, Tomikawa, Nakano & Min, 2020**
–	Pereopod 6, anterior lobe of coxa with 5 robust setae ventrally. Uropods 1–2 outer ramus without robust setae on medial margin	***P. crassus* sp. nov.**

## Supplementary Material

XML Treatment for Pseudocrangonyx concavus

XML Treatment for Pseudocrangonyx gracilipes

XML Treatment for Pseudocrangonyx crassus

XML Treatment for Pseudocrangonyx minutus

XML Treatment for Pseudocrangonyx villosus
